# Dipeptidyl Peptidase 4 (DPP4) Exacerbates Osteoarthritis Progression in an Enzyme‐Independent Manner

**DOI:** 10.1002/advs.202410525

**Published:** 2024-12-16

**Authors:** Xinyu Li, Zhao Zhang, Wenyu Jiang, Yucan Ju, Weihua Guo, Zeyu Huang

**Affiliations:** ^1^ Department of Orthopaedic Surgery Orthopaedic Research Institute West China Hospital West China Medical School Sichuan University Chengdu 610041 China; ^2^ Department of Immuno‐Oncology Beckman Research Institute at City of Hope National Medical Center Duarte 91010 USA

**Keywords:** DPP4, mitochondrial dynamics, MYH9, osteoarthritis, senescence

## Abstract

Chondrocyte senescence is a key driver of osteoarthritis (OA). Mitochondrial dysfunction and oxidative stress can induce chondrocyte senescence. However, the specific mechanisms by which senescence contributes to OA progression are not fully understood. Here, it is attested that Dipeptidyl peptidase 4 (DPP4) is significantly upregulated in osteoarthritic chondrocytes in both humans and mice. DPP4 promotes oxidative stress and cellular senescence in chondrocytes through excessive mitochondrial fission in an enzyme‐independent manner. Intra‐articular injection of adeno‐associated virus 2 to upregulate DPP4 in chondrocytes promotes post‐traumatic and aging‐induced OA in mice in an enzyme‐independent manner. Mechanistically, DPP4 competitively binds to Myosin heavy chain 9 (MYH9), interfering with its E3 ubiquitin ligase Carboxyl terminus of Hsc70‐interacting protein (CHIP), and thereby upregulates MYH9 expression. Finally, a small molecule, 4,5‐Dicaffeoylquinic acid is identified, which disrupts the interaction between DPP4 and MYH9, thereby ameliorating post‐traumatic and aging‐induced OA in mice caused by DPP4 upregulation. The study indicates that the non‐enzymatic activity of DPP4 is a promising target for OA treatment.

## Graphical abstract

In normal conditions, the E3 ubiquitin ligase Carboxyl terminus of Hsc70‐interacting protein (CHIP) binds to and promotes the ubiquitination of Myosin heavy chain 9 (MYH9). The balance between mitochondrial fusion and fission is maintained. In osteoarthritis, Dipeptidyl peptidase 4 (DPP4) competitively binds to MYH9, preventing its ubiquitination. MYH9 leads to excessive mitochondrial fission via the dynamin‐related protein 1 (DRP1)/actin axis.

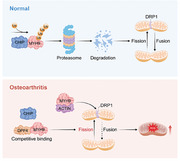

## 1 Introduction

Osteoarthritis (OA) is a leading cause of pain and disability worldwide, impacting 595 million individuals globally.^[^ ^1^ ^]^ Considering the aging of the world's population and the epidemic of obesity, OA has emerged as a substantial public health concern.^[^ ^1^, ^2^ ^]^ Currently, the management of this disease is mainly focused on symptom relief, with joint replacement as an option in more severe cases.^[^ ^3^ ^]^ Disease‐modifying osteoarthritis drugs (DMOADs) are currently unavailable due to the limited understanding of the pathogenesis of OA.^[^ ^4^ ^]^

Cellular senescence, marked by a permanent halt in the cell cycle, has recently been identified as a crucial factor in the development of OA.^[^ ^5^ ^]^ Aging chondrocytes exhibit changes in morphology, function, and metabolism, producing senescence‐associated secretory phenotype (SASP) factors.^[^ ^6^ ^]^ The SASP recruits immune cells, creating an inflammatory environment that degrades the extracellular matrix (ECM) and accelerates cartilage deterioration.^[^ ^7^ ^]^ Mitochondrial dysfunction is closely linked to chondrocyte senescence in OA.^[^ ^8^ ^]^ This dysfunction results in excessive production of reactive oxygen species (ROS), leading to oxidative stress and DNA damage.^[^ ^9^ ^]^ These changes activate the p53 pathway, causing cell cycle arrest and cellular senescence.^[^ ^10^ ^]^ The ensuing cellular senescence exacerbates mitochondrial dysfunction, creating a vicious cycle that ultimately contributes to the progression of OA.^[^ ^11^ ^]^

Dipeptidyl peptidase 4 (DPP4) is well known for its ability to cleave glucagon‐like peptide‐1, which is present both as a membrane protein and in a soluble form^[^ ^12^ ^]^ and plays an important role in cellular senescence. DPP4 was initially discovered to be selectively expressed on the surface of senescent fibroblasts.^[^ ^13^ ^]^ Recent studies have revealed that DPP4 is also highly expressed in aged chondrocytes and the synovial fluid of patients with OA.^[^ ^14^ ^]^ Sitagliptin, a DPP4 enzyme inhibitor, can prevent the development of destabilization of the medial meniscus (DMM)‐induced OA in rats.^[^ ^14b^ ^]^ Despite these findings, the specific role of DPP4 in chondrocyte senescence and its underlying molecular mechanisms in OA remain largely unexplored.

In the present study, we have identified DPP4 as the most prominently expressed molecule in the DPP family associated with OA. Overexpression of DPP4 in chondrocytes aggravates DMM‐induced and aging‐induced OA in mice. DPP4 inhibits the ubiquitination and degradation of Myosin heavy chain 9 (MYH9) without relying on its enzymatic activity, leading to mitochondrial dysfunction and cell aging. Moreover, reducing MYH9 levels with 4,5‐Dicaffeoylquinic acid (4,5‐diCQA) can mitigate OA symptoms in mice induced by DMM surgery and aging. Thus, DPP4 plays a key role in OA pathogenesis in an enzyme‐independent manner and 4,5‐diCQA may have significant potential as a DMOAD.

## 2 Results

### 2.1 DPP4 is Elevated in Chondrocytes in OA

**Figure 1:**
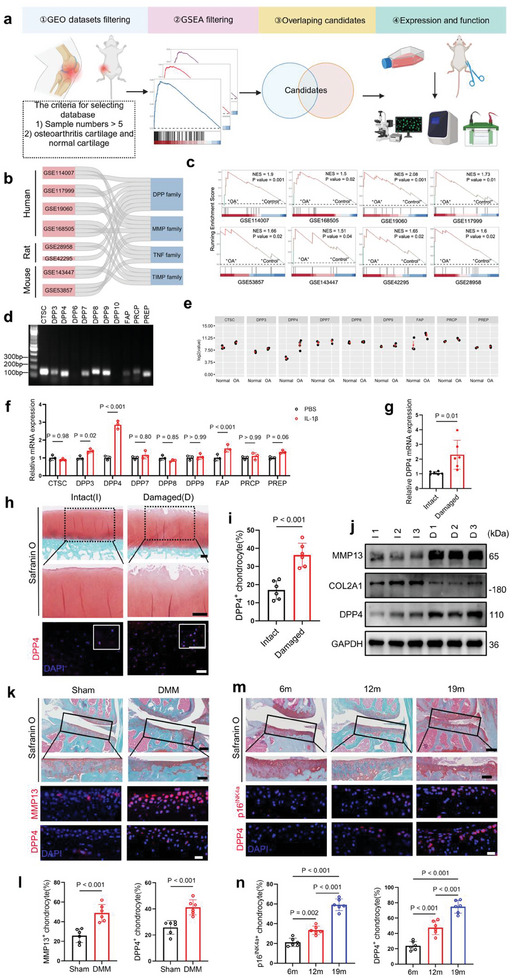
DPP4 is upregulated in osteoarthritic cartilage. a) Workflow for the discovery of DPP4 in OA. GSEA analysis was performed on eight RNA‐seq datasets comparing OA and none‐OA cartilage from the GEO database. Gene families present in each gene set were identified as intersecting gene families. Subsequent in vitro and in vivo experiments were conducted to validate the DPP family. b) The Sankey diagram shows intersecting gene families from RNA‐seq data of human, rat, and mouse. The image was generated by identifying the intersecting gene families enriched from the DEGs in each RNA‐seq dataset. c) GSEA analysis of the DPP family members from different RNA‐seq datasets. d) Agarose gel electrophoresis displays the expression of various genes from the DPP family in C28/I2 human chondrocytes. e) Relative expression levels of different DPP family genes in RNA‐seq data of human OA (*n =* 4) versus healthy (*n =* 3) cartilage (GSE 168505). f RT‐qPCR analysis of the expression of different DPP family genes in primary chondrocytes treated with PBS or IL‐1β for 24 h using *Gapdh* as the reference gene (*n =* 3, biologically independent samples). g) RT‐qPCR analysis of *DPP4* expression in intact and damaged cartilage regions from the same OA patient (*n =* 6, biologically independent samples). h) Safranin‐O/fast green staining (upper images, scale bar = 500 µm) and IF (lower images, scale bar = 50 µm) of intact and damaged cartilage from the same OA patient. i) Quantitative analysis of the proportion of DPP4‐positive cells in the IF results from (h) (*n =* 6, biologically independent samples). j) Protein expression of MMP13, COL2A1, DPP4, and GAPDH in intact and damaged cartilage from the same patient (*n =* 3, biologically independent samples). k) Safranin‐O/fast green staining (scale bar = 250 µm) and IF (scale bar = 20 µm) analysis of knee joints from Sham and DMM surgery mice. l) Quantitative analysis of the proportion of MMP13/DPP4‐positive cells in the IF results from (k) (*n =* 6, biologically independent samples). m) Safranin‐O/fast green staining (scale bar = 250 µm) and IF (scale bar = 20 µm) analysis of knee joints from 6‐, 12‐, and 19‐month‐old mice. n) Quantitative analysis of the proportion of p16^INK4a^/DPP4‐positive cells in the IF results from (m) (*n =* 6, biologically independent samples). Two‐way analysis of variance (ANOVA) followed by Sidak correction for multiple comparisons is used for (f). Two‐tailed t‐tests are used for (g, i, and l) panels. One‐way analysis of variance (ANOVA) followed by Sidak correction for multiple comparisons is used for (n). Quantitative data are shown as mean ± s.d. Exact p‐values are shown in the figures.

To identify potential therapeutic targets for OA, we analyzed published RNA‐sequencing (RNA‐seq) data from cartilage samples of OA patients, as well as OA‐induced rats and mice (**Figure** **1**a). Our study included four human datasets, two rat datasets, and two mouse datasets. Using gene set enrichment analysis (GSEA) comparing OA and non‐OA cartilages, we intersected the data to identify candidate protein families. Our results showed that differentially expressed genes (DEGs) in the DPP family are highly conserved in OA cartilage across humans, rats, and mice. Additionally, we detected several well‐known OA‐related families, including matrix metalloproteinases (MMP),^[^ ^15^ ^]^ tumor necrosis factor (TNF),^[^ ^16^ ^]^ and tissue inhibitors of metalloproteinases (TIMP),^[^ ^17^ ^]^ confirming the reliability of our analysis (Figure 1b,c). The DPP family comprises a group of proteolytic enzymes, including DPP3, DPP4, DPP6, DPP7, DPP8, DPP9, DPP10, cathepsin C (CTSC), prolyl carboxypeptidase (PRCP), fibroblast activation protein α (FAP), and prolyl oligopeptidase (PREP).^[^ ^18^ ^]^ These enzymes play crucial roles in various biological processes such as metabolism, immune response, and protein degradation. They are characterized by their ability to cleave dipeptides from the N‐terminus of polypeptides.^[^ ^19^ ^]^ We amplified Complementary DNA (cDNA) from human chondrocytes using polymerase chain reaction (PCR) assay, and agarose gel electrophoresis results confirmed the expression of *DPP3*, *DPP4*, *DPP7*, *DPP8*, *DPP9*, *CTSC*, *FAP*, *PRCP*, and *PREP* genes in human chondrocytes (Figure 1d). Consistently, RNA‐seq data (GSE168505) from both OA and non‐OA cartilage indicated the expression of these nine DPP family genes in chondrocytes. Notably, *DPP4* exhibited the most significant differential expression between normal and OA cartilage (Figure 1e). PCR with reverse transcription (RT‐qPCR) confirmed a significant increase of *Dpp4* expression in primary mouse chondrocytes treated by Interleukin‐1β (IL‐1β), a pro‐inflammatory cytokine linked to OA (Figure 1f; Figure S1a, Supporting Information).^[^ ^20^ ^]^ Likewise, IL1‐β can also raise the levels of DPP4 protein (Figure S1b, Supporting Information).

We differentiated intact (I) and damaged (D) areas of articular cartilage in the same OA patient based on morphological and histological characteristics (Figure S1c, Supporting Information). RT‐qPCR analysis revealed significantly higher *DPP4* mRNA expression in damaged areas compared to intact areas (Figure 1g). Additionally, Immunofluorescence (IF) analysis demonstrated elevated DPP4 protein levels in damaged cartilage compared to intact cartilage (Figure 1h,i). Western blot analysis of cartilage tissues from three different OA patients showed that, in damaged areas, COL2A1 (anabolic marker) expression was downregulated, while MMP13 (catabolic marker) and DPP4 expressions were upregulated (Figure 1j).

Then we performed the DMM model in 10‐week‐old mice, collected knee joints eight weeks later, and conducted Safranin‐O/fast green staining to assess the success of the model (Figure 1k). IF results indicated that DPP4 expression in chondrocytes of OA mice was elevated (Figure 1k,l). Furthermore, we examined DPP4 expression in the knee cartilage of mice aged 6, 12, and 19 months. Results showed a gradual increase in DPP4 expression with age, accompanied by an increase in the cell senescence marker p16^INK4a^ (Figure 1m,n).^[^ ^21^ ^]^ Additionally, western blot and IF analysis confirmed that DPP4 expression was increased in aged primary mouse chondrocytes (Figure S1d,e, Supporting Information). In summary, these results collectively demonstrated that DPP4 expression is elevated in chondrocytes associated with OA.

### 2.2 DPP4 Overexpression Activates Oxidative Stress and Aging Pathways

**Figure 2:**
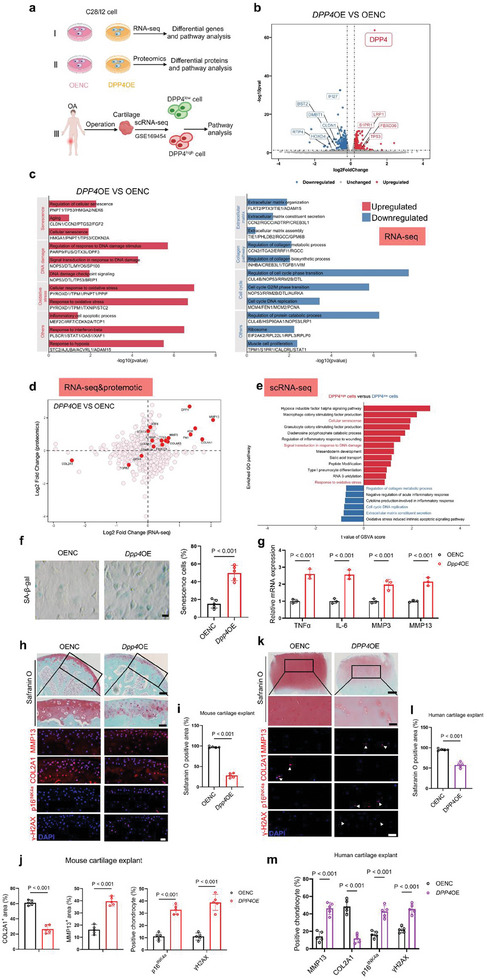
DPP4 promotes oxidative stress and senescence in chondrocytes. a) Schematic diagram of the experimental design. RNA‐seq and proteomics were performed on C28/I2 cells transfected with either OENC or *DPP4*OE plasmids, followed by differential pathway analysis. ScRNA‐seq of cartilage from OA patients in public databases were clustered based on high and low DPP4 expression, and pathway analysis was conducted on these clusters. b) Volcano plot of RNA‐seq data from C28/I2 cells transfected with *DPP4*OE (overexpression) or OENC plasmids (| log_2_ fold change | >0.5, FDR < 0.05).c) Bar plots show representative Gene Ontology terms and pathways of upregulated and downregulated DEGs between C28/I2 cells transfected with OENC and *DPP4*OE plasmids. d) Combined RNA‐seq and proteomics analysis in C28/I2 cells transfected with OENC and *DPP4*OE plasmids. e) Gene set variation analysis (GSVA) of the scRNA‐seq dataset (GSE169454) comparing DPP4^high^ cells with DPP4^low^ cells from cartilage samples of patients with OA. f) SA‐β‐gal staining and quantification of positively stained cells in primary mouse chondrocytes transfected with OENC and *Dpp4*OE plasmids (scale bar = 20 µm) (*n =* 5, biologically independent samples). g) RT‐qPCR analysis showing the relative expression levels of *Tnfa*, *Il‐6*, *Mmp3*, and *Mmp13* in primary mouse chondrocytes transfected with OENC and *Dpp4*OE plasmids, using *Gapdh* as the reference gene (*n =* 3, biologically independent samples). h) Safranin‐O/fast green staining (upper images, scale bar = 250 µm, lower images, scale bar = 50 µm) and IF analysis (scale bar = 20 µm) of mouse cartilage explants transfected with OENC and *Dpp4*OE lentivirus. i) Quantification of Safranin‐O positive areas in (h) (*n =* 5, biologically independent samples). j) Quantification of MMP13, COL2A1, p16^INK4a^, and γH2AX positive cells or regions in the IF results from (h) (*n =* 5, biologically independent samples). k) Safranin‐O/fast green staining (upper images, scale bar = 500 µm, lower images, scale bar = 100 µm) and IF analysis (scale bar = 50 µm) of human cartilage explants transfected with OENC and *DPP4*OE lentivirus. l) Quantification of Safranin‐O positive areas in (k) (*n =* 5, biologically independent samples). m) Quantification of MMP13, COL2A1, p16^INK4a^, and γH2AX positive cells in the IF results from (k) (*n =* 5, biologically independent samples). Two‐way analysis of variance (ANOVA) followed by Sidak correction for multiple comparisons is used for (g, j, and m). Two‐tailed t‐tests are used for (f, i, j, and l) panels. Quantitative data are shown as mean ± s.d. Exact p‐values are shown in the figures.

To investigate the effects of DPP4 upregulation on chondrocyte transcription and protein expression, we overexpressed DPP4 in C28/I2 chondrocytes (Figure S1f, Supporting Information) and conducted RNA‐seq and 4D‐FastDIA quantitative proteomic analysis (**Figure** **2**a–d). Compared to the control group (OENC), we identified 308 upregulated and 285 downregulated DEGs in DPP4 overexpressing chondrocytes (*DPP4*OE) by RNA‐seq analysis (Figure 2b, | log_2_ fold change | >0.5, false discovery rate (FDR)<0.05). RNA‐seq analysis revealed that DPP4 upregulation increased the expression of genes related to oxidative stress and cell senescence while reducing the expression of genes involved in ECM maintenance, collagen synthesis, and cell cycle transition (Figure 2c; Figures S2–S4, Supporting Information). Furthermore, the combined RNA‐seq and proteomics analysis of *DPP4*OE versus OENC highlighted several common targets, among which, upregulated indicators included catabolism‐related gene *MMP3* and *MMP13*, and the aging‐related gene *CDKN1A* (encoding p21) and *TP53* (encoding p53), while downregulated gene included *COL2A1* and *GPX4* (Figure 2d).

To analyze the relative abundance and distribution of *DPP4* transcripts in various chondrocyte subpopulations, we utilized single‐cell sequencing (scRNA‐seq) data from GSE169454. Similar to a recent study,^[^ ^22^ ^]^ unbiased clustering based on known cell‐specific markers identified 7 distinct cell clusters (Figure S5a–c, Supporting Information). We categorized chondrocytes into subpopulations with high and low DPP4 expression based on the average DPP4 expression within each cluster (Figure S5d–f, Supporting Information). The enrichment score of DPP4^high^ cells in the scRNA‐seq OA data was significantly higher than that of DPP4^low^ cells for pathways related to oxidative stress and cellular senescence (Figure 2e).

We overexpressed DPP4 in primary mouse chondrocytes and performed SA‐β‐gal staining, RT‐qPCR analysis, and western blot assay (Figure S6a, Supporting Information). These results showed that DPP4‐overexpressing cells had significantly more positive staining compared to the control group, indicating increased cell senescence (Figure 2f). Additionally, DPP4 overexpression led to the upregulation of multiple SASPs (Figure 2g). Western blot results demonstrated that DPP4 overexpression upregulated p16^INK4a^, p21, and p53, as well as the catabolic molecules COX2 and MMP13, while the anabolic molecules COL2A1 and SOX9 were downregulated (Figure S6b, Supporting Information). In contrast, the knockdown of DPP4 alleviates the IL‐1β‐mediated upregulation of p16^INK4a^, p21, p53, COX2, and MMP13, and promotes the upregulation of COL2A1 and SOX9 (Figure S6c,d, Supporting Information).

Subsequently, we used lentivirus to overexpress DPP4 in human and mouse cartilage explants. The successful transfection was confirmed by the IF assay (Figure S6e, Supporting Information). Safranin‐O/fast green staining revealed that DPP4 overexpression resulted in a decrease in the cartilage matrix compared to the control group (Figure 2h,i,k,l). Furthermore, IF assay indicated that DPP4 overexpression upregulated MMP13, p16^INK4a^, and γH2AX (DNA damage marker), while downregulated COL2A1 (Figure 2h,k,j,m). Therefore, these results suggested that DPP4 overexpression may disrupt the balance between anabolism and catabolism by engaging oxidative stress‐related and senescence‐related pathways.

### 2.3 DPP4 Disrupts Mitochondrial Dynamics, Leading To Cellular Oxidative Stress and Senescence

**Figure 3:**
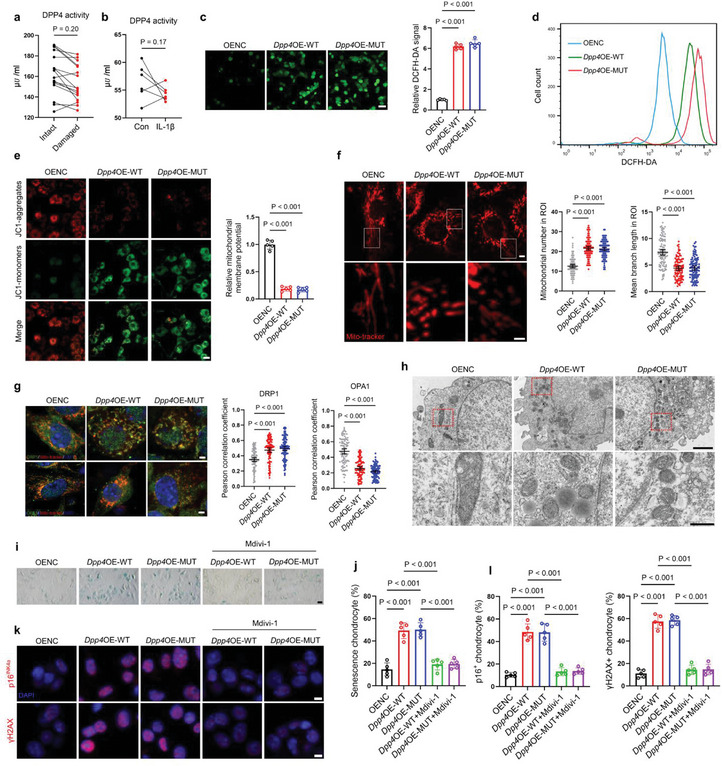
DPP4 promotes mitochondrial fission in chondrocytes. a) Enzyme activity of DPP4 in intact and damaged cartilage from OA patients (*n =* 16, biologically independent samples). b) Enzyme activity of DPP4 in primary mouse chondrocytes treated with vehicle or 10 ng mL^−1^ IL‐1β for 24 h (*n =* 6, biologically independent samples). c) IF assay and quantification of DCFH‐DA signal in primary mouse chondrocytes transfected with OENC, *Dpp4*OE‐WT, or *Dpp4*OE‐MUT plasmids (scale bar = 50 µm) (*n =* 5, biologically independent samples). d) Flow cytometry detection of DCFH‐DA signal in primary mouse chondrocytes transfected with OENC, *Dpp4*OE‐WT, or *Dpp4*OE‐MUT plasmids. e) IF assay and quantification of mitochondrial membrane potential in primary mouse chondrocytes transfected with OENC, *Dpp4*OE‐WT, or *Dpp4*OE‐MUT plasmids (scale bar = 20 µm) (*n =* 5, biologically independent samples). f) Mitotracker staining and quantitative analysis of the mitochondrial number and mean branch length in regions of interest (ROI) in primary mouse chondrocytes transfected with OENC, *Dpp4*OE‐WT, or *Dpp4*OE‐MUT plasmids (upper images, scale bar = 5 µm, lower images, scale bar = 2 µm) (*n =* 5, biologically independent samples, 100 cells per group). For each sample, cells were randomly selected, and a 15Í20 µm region was chosen as the ROI within each cell. Quantitative analysis was performed using the mitochondrial analyzer plugin in Image J. g) IF and quantitative analysis of DRP1/OPA1 and mitochondrial colocalization in primary mouse chondrocytes transfected with OENC, *Dpp4*OE‐WT, or *Dpp4*OE‐MUT plasmids (scale bar = 2 µm) (*n =* 5, biologically independent samples, 100 cells per group). Colocalization quantitative analysis was performed using the Coloc 2 plugin in Image J. h) Transmission electron microscopy images of primary mouse chondrocytes transfected with OENC, *Dpp4*OE‐WT, or *Dpp4*OE‐MUT plasmids (upper images, scale bar = 2 µm, lower images, scale bar = 0.5 µm). i) SA‐β‐gal staining of primary mouse chondrocytes transfected with OENC, *Dpp4*OE‐WT, or *Dpp4*OE‐MUT plasmids, with or without Mdivi‐1 (10 µм) (scale bar = 20 µm). j) Quantification of SA‐β‐gal positive cells in (i) (*n =* 5, biologically independent samples). k) IF analysis of primary mouse chondrocytes transfected with OENC, *Dpp4*OE‐WT, or *Dpp4*OE‐MUT plasmids, with or without Mdivi‐1 (10 µм). l) Quantification of p16^INK4a^ or γH2AX positive cells in the IF results from (k) (scale bar = 5 µm) (*n =* 5, biologically independent samples). Two‐tailed t‐tests are used for (a and b). One‐way analysis of variance (ANOVA) followed by Sidak correction for multiple comparisons is used for (c, e, f, g, j, and l). Quantitative data are shown as mean ± s.d. Exact p‐values are shown in the figures.

Previous studies have shown that DPP4 can cause mitochondrial dysfunction through its non‐enzymatic functions.^[^ ^23^ ^]^ To determine whether DPP4 causes mitochondrial dysfunction via its enzymatic or non‐enzymatic functions, leading to cellular oxidative stress and aging, we assessed DPP4 enzymatic activity in intact and damaged cartilage from OA patients. The results indicated no significant difference in DPP4 enzyme activity between intact and damaged areas (**Figure** **3**a). Similarly, IL1β did not increase DPP4 enzyme activity in primary mouse chondrocytes (Figure 3b). These findings suggest that, unlike DPP4 mRNA or protein levels, DPP4 enzyme activity is not elevated in osteoarthritic cartilage.

Next, we identified the active site of the DPP4 enzyme and constructed a mutant plasmid with an inactive enzyme site(S624A).^[^ ^23^, ^24^ ^]^ We then transfected primary mouse chondrocytes with control plasmid (OENC), *Dpp4* wild‐type plasmid (*Dpp4*OE‐WT), and DPP4 enzyme active site mutant plasmid (*Dpp4*OE‐MUT), respectively. Chondrocytes transfected with *Dpp4*OE‐WT and *Dpp4*OE‐MUT exhibited significantly elevated levels of ROS, as determined by the redox‐sensitive fluorescent indicator DCFH‐DA, which measures intracellular H_2_O_2_ levels (Figure 3c,d). Additionally, the mitochondrial membrane potential in chondrocytes transfected with either *Dpp4*OE‐WT or *Dpp4*OE‐MUT plasmids significantly decreased (Figure 3e). Compared to the OENC group, the mitochondria in the *Dpp4*OE‐WT and *Dpp4*OE‐MUT groups exhibited more numerous and shorter branches, indicating an increase in mitochondrial fission (Figure 3f). IF results showed a significant upregulation in the co‐localization of mitochondria and dynamin‐related protein 1 (DRP1) (a mitochondrial fission protein) in both *Dpp4*OE‐WT and *Dpp4*OE‐MUT groups. Conversely, the co‐localization between mitochondria and OPA1 (a mitochondrial fusion protein) was significantly downregulated, suggesting that mitochondrial fission surpassed mitochondrial fusion (Figure 3g). Similarly, transmission electron microscopy revealed an increase in the number of mitochondria and a reduction in their size following *Dpp4* overexpression, regardless of whether it was the wild‐type or mutant type (Figure 3h). Under physiological conditions, mitochondrial fission and fusion in chondrocytes maintain a stable balance. However, in OA, chondrocytes experience more frequent mitochondrial fission, resulting in mitochondrial network fragmentation and dysfunction.^[^ ^25^ ^]^

To determine whether DPP4 induces oxidative stress and cell senescence by inducing excessive mitochondrial fission, we added Mdivi‐1 (DRP1 inhibitor) to DPP4‐overexpressing chondrocytes and performed SA‐β‐gal staining and IF assay. The results showed that Mdivi‐1 could reverse oxidative stress and cell senescence in *Dpp4*OE‐WT and *Dpp4*OE‐MUT groups (Figure S6f, Supporting Information; Figure 3i–l). These data demonstrated that DPP4 disrupts mitochondrial dynamics in chondrocytes in an enzyme‐independent manner, thereby inducing oxidative stress and cellular senescence.

### 2.4 Overexpression of DPP4 in Chondrocytes Aggravates Mice OA Progression Induced by DMM and Aging

**Figure 4:**
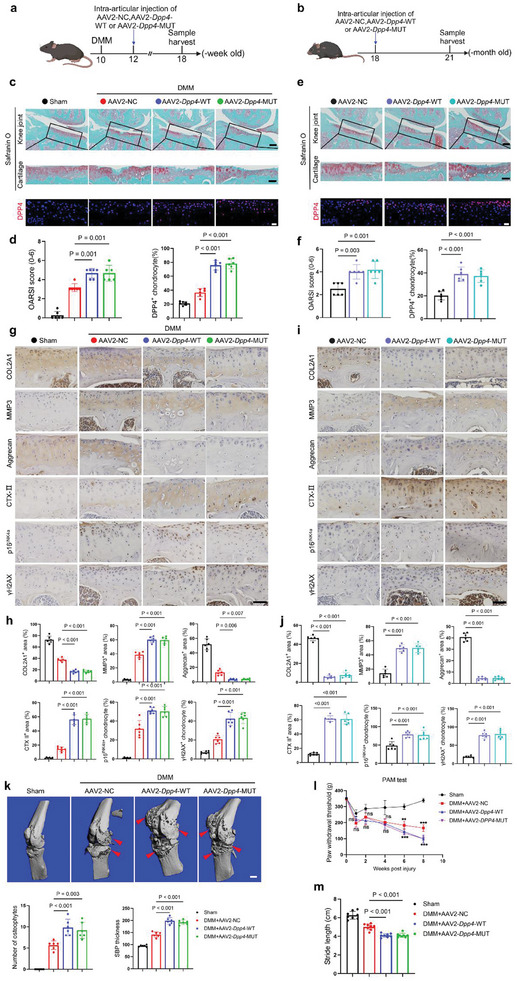
DPP4 promotes OA progression in mice induced by DMM and aging. a) Schematic diagram of the experimental design: 10‐week‐old mice underwent Sham or DMM surgery. Two weeks after surgery, the DMM group received intra‐articular injections of AAV2‐NC, AAV2‐*Dpp4*‐WT, or AAV2‐*Dpp4*‐MUT. Knee joints were collected 6 weeks later. b) Schematic diagram of the experimental design: 18‐month‐old mice received intra‐articular injections of AAV2‐NC, AAV2‐*Dpp4*‐WT, or AAV2‐*Dpp4*‐MUT. Knee joints were collected 3 months later. c) Safranin‐O/fast green staining (scale bar = 250 µm) and IF (scale bar = 20 µm) analysis of knee joints from Sham, DMM+AAV2‐NC, DMM+AAV2‐*Dpp4*‐WT, and DMM+AAV2‐*Dpp4*‐MUT groups. d) OARSI scores and quantitative analysis of the number of DPP4‐positive cells from (c) (*n =* 6 mice per group). e) Safranin‐O/fast green staining (scale bar = 250 µm) and IF (scale bar = 20 µm) analysis of knee joints from AAV2‐NC, AAV2‐*Dpp4*‐WT, and AAV2‐*Dpp4*‐MUT groups. f) OARSI scores and quantitative analysis of the number of DPP4‐positive cells from (e) (*n =* 6 mice per group). g) IHC analysis of knee joints from Sham, DMM+AAV2‐NC, DMM+AAV2‐*Dpp4*‐WT, and DMM+AAV2‐*Dpp4*‐MUT groups (scale bar = 50 µm). h) Quantitative analysis of results from (g) (*n =* 6 mice per group). i) IHC analysis of knee joints from AAV2‐NC, AAV2‐*Dpp4*‐WT, and AAV2‐*Dpp4*‐MUT groups (scale bar = 50 µm). j) Quantitative analysis of results from (i) (*n =* 6 mice per group). k) Micro‐CT analysis of knee joints from Sham, DMM+AAV2‐NC, DMM+AAV2‐*Dpp4*‐WT, and DMM+AAV2‐*Dpp4*‐MUT groups, including quantitative analysis of osteophyte number and subchondral bone plate (SBP) thickness (scale bar = 1000 µm) (*n =* 6 mice per group). l) Knee joint pain assessment in Sham, DMM+AAV2‐NC, DMM+AAV2‐*Dpp4*‐WT, and DMM+AAV2‐*Dpp4*‐MUT groups (*n =* 8 mice per group). m) Stride length measurement in Sham, DMM+AAV2‐NC, DMM+AAV2‐*Dpp4*‐WT, and DMM+AAV2‐*Dpp4*‐MUT groups (*n =* 8 mice per group). One‐way analysis of variance (ANOVA) followed by Sidak correction for multiple comparisons is used for (d, f, h, j, k, and m). Two‐way analysis of variance (ANOVA) followed by Sidak correction for multiple comparisons is used for (l). Quantitative data are shown as mean ± s.d. Exact p‐values are shown in the figures. ***p < 0.001, **p < 0.01, NS, no significance.

To investigate the role of DPP4 in the OA mice model, we injected adeno‐associated viruses 2 (AAV2) containing AAV2‐NC, AAV2‐*Dpp4*‐WT, or AAV2‐*Dpp4*‐MUT into the mice knee articular cavity. For DMM‐induced OA, 10‐week‐old mice received an intra‐articular injection two weeks post‐DMM, and samples were collected six weeks later. For aging‐induced OA, 18‐month‐old mice received an intra‐articular injection, and samples were collected three months later (**Figure** **4**a,b). The infection of AAV2 in chondrocytes was confirmed by detecting the expression of Green fluorescent protein (GFP) through IF analysis (Figure S7a, Supporting Information). Morphological and IF analyses revealed that mice in the AAV2‐*Dpp4*‐WT and AAV2‐*Dpp4*‐MUT groups had higher Osteoarthritis Research Society International (OARSI) scores and increased DPP4 expression compared to the AAV‐NC group in both DMM‐induced and aging‐induced OA models (Figure 4c–f). Additionally, there were no significant differences in morphology or DPP4 expression between the AAV2‐*Dpp4*‐WT and AAV2‐*Dpp4*‐MUT groups. Additionally, the upregulation of DPP4 leads to more severe synovitis in mice induced by both DMM and aging (Figure S7b,c, Supporting Information). Immunohistochemistry (IHC) results showed that mice in the AAV2‐*Dpp4*‐WT and AAV2‐*Dpp4*‐MUT groups had lower levels of cartilage matrix components, including COL2A1 and Aggrecan. Conversely, these groups exhibited higher levels of MMP3, the cartilage degradation component CTX‐II, and senescence‐related molecules such as p16^INK4a^ and γH2AX (Figure 4g–j). In addition, transmission electron microscopy results of mouse knee cartilage tissue showed that compared to AAV2‐NC, both AAV2‐*Dpp4*‐WT and AAV2‐*Dpp4*‐MUT led to an increase in mitochondrial fission in chondrocytes (Figure S7d, Supporting Information). Consistent with the histological analysis, micro‐CT analysis of non‐decalcified knee joints from DMM‐induced OA mice showed that the AAV2‐*Dpp4*‐WT and AAV2‐*Dpp4*‐MUT groups exhibited more osteophyte formation and subchondral bone sclerosis than the AAV2‐NC group (Figure 4k). Given that pain is the most prominent symptom of OA, we used a pressure application measurement (PAM) device to assess local joint pain in DMM‐induced OA mice.^[^ ^26^ ^]^ Mice in the AAV2‐*Dpp4*‐WT and AAV2‐*Dpp4*‐MUT groups had lower pain thresholds compared to those in the AAV2‐NC group, indicating poorer pain tolerance (Figure 4l). Gait analysis results showed that stride length in mice shortened after DMM surgery. Additionally, the stride length in the AAV2‐*Dpp4*‐WT and AAV2‐*Dpp4*‐MUT groups was shorter than that in the AAV2‐NC group, indicating that upregulation of DPP4 can cause motor dysfunction in mice (Figure 4m; Figure S7e, Supporting Information). These results collectively indicate that upregulation of DPP4 exacerbates both DMM‐induced and aging‐induced OA in mice through an enzyme‐independent mechanism.

### 2.5 DPP4 Binds to MYH9 and Upregulates its Expression in an Enzyme‐Independent Manner

**Figure 5:**
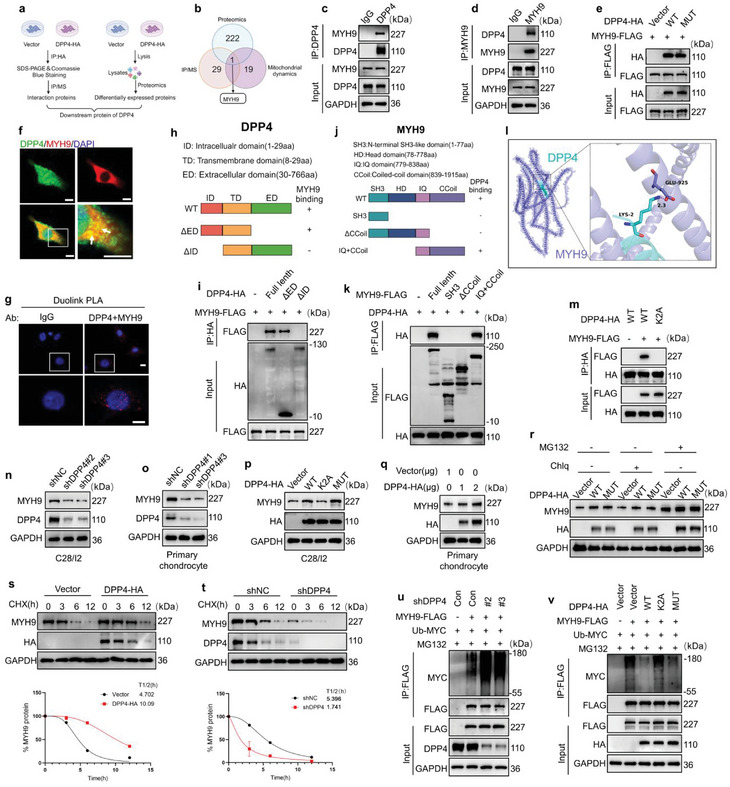
DPP4 binds and downregulates MYH9 expression. a) The schematic diagram of identifying MYH9 as a DPP4 binding target. IP‐MS and proteomics analyses were performed on C28/I2 cells transfected with either vector or DPP4‐HA plasmids. The intersecting proteins from these analyses were then identified and validated. b) Venn diagram showing the intersection of proteomics, IP‐MS, and mitochondrial dynamics‐related proteins. c,d) Reciprocal Co‐IP analysis showing the endogenous interaction between DPP4 and MYH9 in C28/I2 cells (*n =* 3). e) IP analysis shows the binding between MYH9‐FLAG and DPP4‐HA‐WT or DPP4‐HA‐MUT in C28/I2 cells (*n =* 3). f) IF analysis showing colocalization of DPP4 (green) and MYH9 (red) in C28/I2 cells (scale bar = 10 µm) (*n =* 3). g) PLA analysis showing the binding of DPP4 and MYH9 in C28/I2 cells (scale bar = 10 µm) (*n =* 3). h) Schematic diagram of DPP4 domains and truncation mutations. i) Wildtype and different truncation mutations of DPP4‐HA were transfected into C28/I2 cells along with MYH9‐FLAG. DPP4‐HA was immunoprecipitated with HA beads (*n =* 3). j) Schematic diagram of MYH9 domains and truncation mutations. k) Wildtype and different truncation mutations of MYH9‐FLAG were transfected into C28/I2 cells along with DPP4‐HA. MYH9‐FLAG was immunoprecipitated with FLAG beads (*n =* 3). l) Molecular docking showing the electrostatic interaction between DPP4 and MYH9. m) IP analysis shows the binding between MYH9‐FLAG and DPP4‐HA‐WT but not DPP4‐HA‐K2A (*n =* 3). n) Western blot shows that downregulating DPP4 leads to a reduction of MYH9 in (n) C28/I2 cells and o) primary mouse chondrocytes (*n =* 3). p) MYH9 protein expression in C28/I2 cells transfected with DPP4‐HA‐WT, DPP4‐HA‐MUT and DPP4‐HA‐K2A plasmids (*n =* 3). q) Western blot shows that DPP4‐HA upregulates MYH9 in primary mouse chondrocytes (*n =* 3). r) C28/I2 cells with ectopic expression of WT or MUT DPP4‐HA were treated with DMSO, Chlq (10 µм), or MG132 (10 µм) for 8 h (*n =* 3). s,t) C28/I2 cells were transfected with vector or DPP4‐HA and shNC or shDPP4 and treated with CHX (10 µм) for different durations (upper). Relative expression levels of MYH9 protein over time (lower) (*n =* 3). u) Effects of shNC and shDPP4 on the ubiquitination of MYH9‐FLAG in C28/I2 cells analyzed by in vivo ubiquitination assay (*n =* 3). v) Ubiquitination of MYH9‐FLAG in C28/I2 cells with vector, DPP4‐HA‐WT, DPP4‐HA‐K2A, DPP4‐HA‐MUT, and Ub‐MYC transfection (*n =* 3). *n =* 3 replicate independent biological replicates.

To identify the targets of DPP4's non‐enzymatic functions, we performed immunoprecipitation‐Mass spectrometry (IP‐MS) and proteomic analysis on C28/I2 cells transfected with the DPP4‐HA plasmid. This analysis identified 30 DPP4 binding proteins and 223 differentially expressed proteins following DPP4 upregulation (**Figure** **5**a,b). By intersecting these results with 20 mitochondrial dynamics‐related proteins, we identified MYH9 as the only target protein (Figure 5b; Figure S8a,b, Supporting Information). MYH9 has been previously reported to promote mitochondrial fission by regulating the actin network and DRP1 localization.^[^ ^27^ ^]^

To verify the binding relationship between DPP4 and MYH9, we conducted a co‐immunoprecipitation (Co‐IP) analysis in C28/I2 cells. The results showed that DPP4 and MYH9 bind both endogenously and exogenously (Figure 5c,d; Figure S8c,d, Supporting Information). Additionally, IP assay with C28/I2 cells transfected with either the DPP4 enzyme activity site mutant plasmid (DPP4‐MUT) or the wild‐type plasmid (DPP4‐WT) indicated that the binding between DPP4 and MYH9 is independent of DPP4's enzymatic activity (Figure 5e). IF analysis showed co‐localization of DPP4 and MYH9 in C28/I2 cells (Figure 5f). Similarly, a proximity ligation assay (PLA) demonstrated a binding relationship between DPP4 and MYH9 in C28/I2 cells (Figure 5g).

To determine the binding domains of DPP4 and MYH9, we constructed various truncated mutant plasmids and transfected them into C28/I2 cells. Co‐IP results suggested that the intracellular domain of DPP4 (residues 1–29) is necessary for binding to MYH9, while the coiled‐coil domain of MYH9 (residues 839–1915) is required for binding to DPP4 (Figure 5h–k). Molecular docking analysis further indicated a binding interaction between the second lysine of DPP4 and the 925th glutamic acid of MYH9 (Figure 5l). To verify it, we constructed a DPP4 site mutation plasmid (K2A) and transfected it into C28/I2 cells, resulting in a loss of binding to MYH9 (Figure 5m). Then, we knocked down DPP4 expression in primary mouse chondrocytes and C28/I2 cells. The knockdown efficiency of DPP4 was confirmed by western blot analysis (Figure S9a, Supporting Information). It showed that MYH9 expression decreased following DPP4 downregulation (Figure 5n,o). Overexpression of DPP4‐WT or DPP4‐MUT in C28/I2 cells upregulated MYH9 expression, whereas the K2A mutation did not (Figure 5p). In primary mouse chondrocytes, DPP4 upregulated MYH9 expression in a concentration‐dependent manner (Figure 5q). However, DPP4 upregulation did not increase MYH9 mRNA levels, indicating that DPP4 does not regulate MYH9 expression at the transcriptional level (Figure S9b, Supporting Information). Since DPP4 can upregulate MYH9 expression, and MYH9 facilitates mitochondrial fission through DRP1 and actin, we aimed to determine whether the regulation of mitochondrial fission by DPP4 occurs via the MYH9/DRP1/actin axis. We added the DRP1 inhibitor Mdivi‐1 and the actin polymerization inhibitor Cytochalasin D to chondrocytes overexpressing *Dpp4*‐WT and *Dpp4*‐MUT. Results from Mito‐tracker analysis show that both Mdivi‐1 and Cytochalasin D can inhibit the excessive mitochondrial fission caused by the upregulation of DPP4 (Figure S9c–f, Supporting Information).

Considering our results that DPP4 binds to MYH9 without upregulating MYH9 protein expression at the transcriptional level, we speculated that DPP4 affects MYH9 protein degradation via the proteasome or lysosome pathway. To test this hypothesis, we transfected C28/I2 cells with a control plasmid (Vector), DPP4‐WT, or DPP4‐MUT plasmid and treated them with the proteasome pathway inhibitor MG132 or the lysosomal pathway inhibitor Chlq. MYH9 expression was then assessed by western blot analysis. Only MG132 treatment upregulated MYH9 expression in cells and abolished the upregulation of MYH9 expression by DPP4‐WT or DPP4‐MUT (Figure 5r). To determine whether DPP4 affects the protein stability of MYH9, we measured the half‐life of MYH9 protein in cells. C28/I2 cells, with or without DPP4‐HA expression, were treated with the protein synthesis inhibitor cycloheximide (CHX) for different durations before western blot assays. Compared to the control group, DPP4‐HA expression significantly increased the half‐life of MYH9 protein (Figure 5s). Conversely, knocking down DPP4 expression significantly decreased the half‐life of MYH9 protein (Figure 5t). These data indicate that DPP4 stabilizes MYH9 protein by inhibiting its proteasomal degradation. To investigate whether DPP4 regulates the ubiquitination of MYH9, we co‐transfected DPP4 shRNAs, MYH9‐FLAG plasmid, and ubiquitin plasmid (Ub‐MYC) into C28/I2 cells and treated the cells with MG132. IP analysis showed that decreased DPP4 expression upregulated the ubiquitination of MYH9 (Figure 5u). In contrast, transfection with DPP4‐WT and DPP4‐MUT plasmids downregulated MYH9 ubiquitination, while the K2A plasmid failed to reduce MYH9 ubiquitination (Figure 5v). Taken together, these results suggested that DPP4 binds to MYH9 in an enzyme‐independent manner, hindering the degradation of MYH9 protein via the ubiquitin‐proteasome pathway and thereby upregulating MYH9 protein expression.

### 2.6 DPP4 Stabilizes MYH9 by Interrupting CHIP‐Mediated Ubiquitination Degradation of MYH9

**Figure 6:**
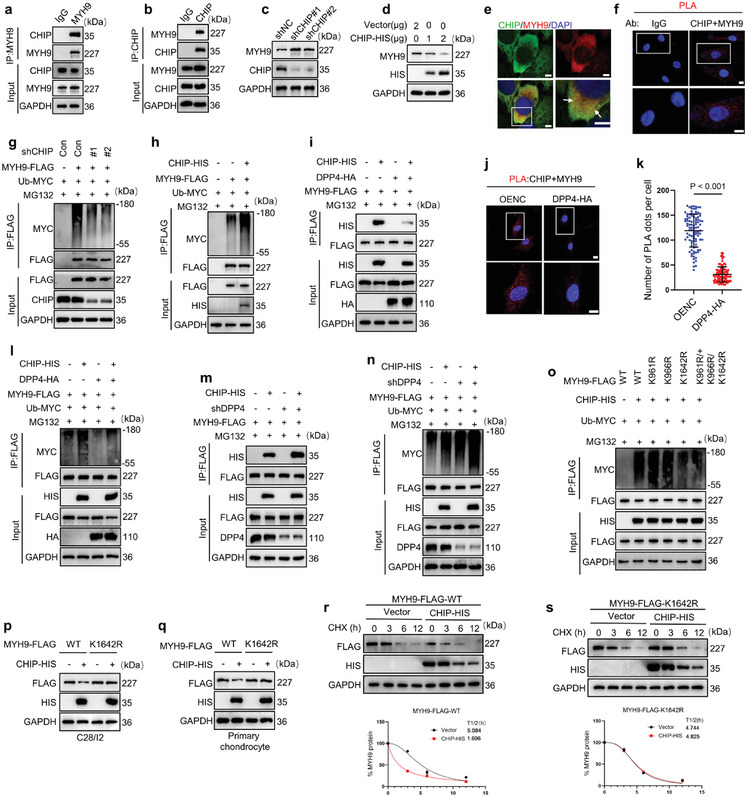
DPP4 interferes with CHIP‐mediated ubiquitination and degradation of MYH9. a,b) Co‐IP analysis shows the endogenous interaction between MYH9 and CHIP in C28/I2 cells. (*n =* 3). c) Downregulation of CHIP expression upregulates MYH9 in C28/I2 cells (*n =* 3). d) CHIP‐HIS downregulates MYH9 expression in C28/I2 cells (*n =* 3). e) IF analysis showing colocalization of MYH9 (red) and CHIP (green) in C28/I2 cells (scale bar = 10 µm) (*n =* 3). f) PLA analysis showing the interaction between MYH9 and CHIP in C28/I2 cells (scale bar = 10 µm) (*n =* 3). IgG was used as a negative control. g) Western blot of ubiquitination levels of MYH9‐FLAG in CHIP knockdown C28/I2 cells (*n =* 3). h) Western blot of ubiquitination levels of MYH9‐FLAG in CHIP‐HIS overexpressing C28/I2 cells (*n =* 3). i) Western blot of MYH9‐FLAG expression in C28/I2 cells overexpressing CHIP‐HIS and DPP4‐HA (*n =* 3). j) PLA analysis of the interaction between MYH9 and CHIP in C28/I2 cells transfected with OENC or DPP4‐HA plasmids (scale bar = 10 µm) (*n =* 3). k) Quantitative analysis from (j) (*n =* 3, 100 cells per group). l) Ubiquitination of MYH9‐FLAG in C28/I2 cells transfected with CHIP‐HIS, DPP4‐HA, MYH9‐FLAG, and Ub‐MYC (*n =* 3). m) In C28/I2 cells with DPP4 knocked down, overexpress CHIP‐HIS and MYH9‐FLAG, then pull down MYH9‐FLAG using FLAG beads and detect CHIP‐HIS expression. (*n =* 3). n) Ubiquitination levels of MYH9‐FLAG in C28/I2 cells transfected with shDPP4, CHIP‐HIS and MYH9‐FLAG plasmids(*n =* 3). o) In vivo ubiquitination assays in C28/I2 cells expressing WT or indicated mutant MYH9‐Flag plasmids(*n =* 3). p) Western blot analysis of MYH9‐FLAG expression in C28/I2 cells transduced with WT or 1642R MYH9‐FLAG vectors along with CHIP‐HIS vectors (*n =* 3). q) Western blot analysis of MYH9‐FLAG expression in primary mouse chondrocytes transduced with WT or 1642R MYH9‐FLAG plasmids along with CHIP‐HIS (*n =* 3). r) Western blot analysis in C28/I2 cells transfected with MYH9‐FLAG‐WT, vector, and CHIP‐HIS and treated with CHX (10 µм) for different time intervals (upper). Relative expression levels of MYH9 protein over time (lower) (*n =* 3). s) Western blot analysis in C28/I2 cells transfected with MYH9‐FLAG‐K1642R, vector, and CHIP‐HIS and treated with CHX (10 µм) for different time intervals (upper). Relative expression levels of MYH9 protein over time (lower) (*n =* 3). Two‐tailed t‐tests are used for (k). *n =* 3 replicate independent biological replicates.

Considering that protein degradation by the proteasome pathway is primarily mediated by E3 ubiquitin ligases, we speculated that DPP4 might affect the degradation of MYH9 through a specific E3 ubiquitin ligase. Previous reports have identified the carboxyl terminus of Hsc70‐interacting protein (CHIP) as the E3 ubiquitin ligase for MYH9.^[^ ^28^ ^]^ Co‐IP assay demonstrated a binding relationship between DPP4 and CHIP in C28/I2 cells (**Figure** **6**a,b). To demonstrate the regulatory effect of CHIP on MYH9, we constructed CHIP shRNAs and an overexpression plasmid CHIP‐HIS and transfected them into C28/I2 cells, respectively. Western blot results showed that downregulating CHIP expression upregulated MYH9 expression (Figure 6c; Figure S9g, Supporting Information). Conversely, overexpressing CHIP downregulated MYH9 expression in a concentration‐dependent manner (Figure 6d). IF analysis showed that MYH9 and CHIP proteins co‐localize in C28/I2 cells (Figure 6e). Similarly, PLA demonstrated a binding relationship between MYH9 and CHIP proteins (Figure 6f). To verify whether CHIP regulates the ubiquitination of MYH9, we co‐transfected shRNAs, MYH9‐FLAG plasmid, and Ub‐MYC plasmid into C28/I2 cells, followed by treatment with MG132. IP analysis showed that downregulating CHIP expression reduced the ubiquitination of MYH9 (Figure 6g). Conversely, overexpression of CHIP in C28/I2 cells increased the ubiquitination of MYH9 (Figure 6h). In summary, these data confirmed that CHIP binds to MYH9 in C28/I2 cells and promotes its ubiquitination.

Since competitive binding of other proteins to E3 ubiquitin ligase substrate proteins can reduce the degradation of those substrate proteins, we speculated that DPP4 might hinder the degradation of MYH9 through this pathway. To verify this hypothesis, we co‐transfected DPP4‐HA, CHIP‐HIS, and MYH9‐FLAG plasmids into C28/I2 cells and treated them with MG132. IP analysis confirmed that the upregulation of DPP4 reduced the binding between MYH9 and CHIP (Figure 6i). Similarly, PLA revealed that overexpression of DPP4 reduced the binding between MYH9 and CHIP (Figure 6j,k). Additionally, upregulation of DPP4 reduced MYH9 ubiquitination induced by CHIP overexpression (Figure 6l). Conversely, downregulation of DPP4 increased the binding between MYH9 and CHIP and upregulated MYH9 ubiquitination induced by CHIP overexpression (Figure 6m,n).

To identify the ubiquitination sites of the MYH9 protein, we performed LC‐MS/MS detection following plasmid transfection and IP assay in C28/I2 cells. The detailed process is shown in Figure S9h (Supporting Information). We identified three potential ubiquitination sites on MYH9: K961, K966, and K1642 (Figures S9i and S10a, Supporting Information). To determine which site is essential for CHIP‐mediated ubiquitination, we constructed plasmids with mutations at these sites (K961R, K966R, K1642R, K961R/K966R/K1642R) by replacing lysine with arginine and transfected them into C28/I2 cells. IP analysis showed that CHIP‐mediated ubiquitination of MYH9 was downregulated only when the K1642 site was mutated (Figure 6o). Additionally, the K1642 site of MYH9 is conserved across different species (Figure S10b, Supporting Information). Compared with the wild‐type MYH9‐FLAG plasmid, CHIP upregulation could no longer downregulate MYH9 expression in C28/I2 cells and primary chondrocytes transfected with the MYH9‐FLAG(K1642R) plasmid (Figure 6p,q). CHIP overexpression significantly decreased the half‐life of wild‐type MYH9, but not the K1642R mutant MYH9 (Figure 6r,s). Together, these data supported that DPP4 and CHIP competitively bind to MYH9, resulting in reduced ubiquitination and elevated levels of MYH9.

### 2.7 4,5‐diCQA Promotes the Ubiquitination and Degradation of MYH9 by Disrupting the Interaction Between DPP4 and MYH9

**Figure 7:**
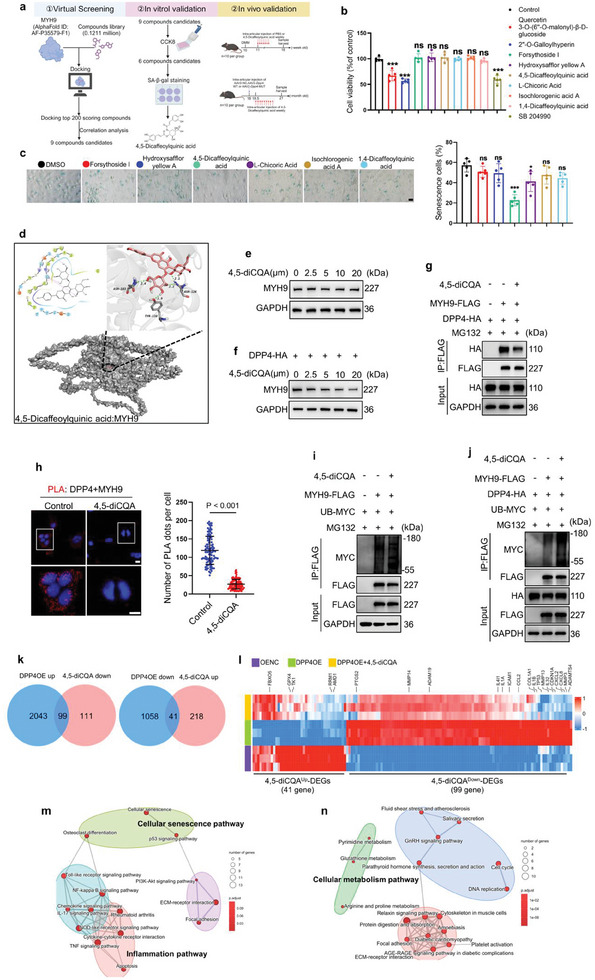
4,5‐diCQA disrupts the interaction between DPP4 and MYH9 and promotes the degradation of MYH9. a) Schematic diagram of the experimental design. Virtual screening was performed to identify small molecule compounds that can bind to human MYH9 protein. Candidates were selected based on binding scores and previous reports. The effects of these compounds on the proliferation of primary mouse chondrocytes were assessed using the CCK8 assay, and their impact on cellular senescence was determined using SA‐β‐Gal staining. The effects of the compounds on OA were then validated in mouse OA models. b) CCK8 assay to assess cell viability of primary mouse chondrocytes treated with nine different drugs (*n =* 5 independent biological replicates). All drugs were used at a concentration of 10 µm. c) SA‐β‐Gal staining analysis to evaluate the effect of six drugs on senescent chondrocytes (left). Quantitative analysis of the proportion of senescent cells (right) (scale bar = 20 µm) (*n =* 5 independent biological replicates). d) The molecular structure of 4,5‐diCQA and a model of the interaction between 4,5‐diCQA and human MYH9. e) Different concentrations of 4,5‐diCQA were added to C28/I2 cells, and MYH9 levels were analyzed by western blot (*n =* 3 independent biological replicates). f) Different concentrations of 4,5‐diCQA were added to DPP4‐overexpressing C28/I2 cells, and MYH9 levels were analyzed by western blot (*n =* 3 independent biological replicates).g) In C28/I2 cells transfected with MYH9‐FLAG and DPP4‐HA plasmids, the addition of 4,5‐diCQA, followed by IP pull‐down of MYH9‐FLAG and western blot analysis to detect DPP4‐HA levels (*n =* 3 independent biological replicates) h) C28/I2 cells were treated with or without 4,5‐diCQA, and the interaction between DPP4 and MYH9 was analyzed using PLA (scale bar = 10 µm), followed by quantitative analysis (*n =* 3 independent biological replicates, 100 cells per group). In normal i) and DPP4‐overexpressing j) C28/I2 cells, 4,5‐diCQA was added or omitted, and an IP assay was conducted to assess the ubiquitination levels of MYH9‐FLAG (*n =* 3 independent biological replicates). k) Identification of DEGs in C28/I2 cells transfected with DPP4OE plasmid compared to OENC plasmid and the effect of 4,5‐diCQA (10 µм) treatment on these DEGs (FDR<0.05). l) Heatmap of the 4,5‐diCQAUp‐DEGs and the 4,5‐diCQADown‐DEGs. m) Pathway analysis of the 4,5‐diCQADown‐DEGs. n) Pathway analysis of the 4,5‐diCQAUp‐DEGs. One‐way analysis of variance (ANOVA) followed by Sidak correction for multiple comparisons is used for (b and c). Two‐tailed t‐tests are used for (h). Quantitative data are shown as mean ± s.d. ***p < 0.001, *p < 0.05, NS, no significance.

To explore potential therapeutic options to mitigate DPP4‐mediated upregulation of MYH9 expression in OA, we conducted virtual screening to identify small molecule compounds that disrupt the interaction between DPP4 and MYH9 protein. By ranking binding scores and screening for drug functions (anti‐inflammatory, anti‐oxidative stress, anti‐aging), we identified nine potential compounds (**Figure** **7**a), among which, six compounds—Forsythoside I, Hydroxysafflor yellow A, 4,5‐diCQA, L‐Chicoric Acid, Isochlorogenic acid A, and 1,4‐Dicaffeoylquinic acid—showed no adverse effects on primary chondrocyte proliferation, as determined by the Cell Counting Kit‐8 (CCK8) assay (Figure 7b). We then continuously passaged primary mouse chondrocytes to the sixth generation and treated them with these six candidate compounds. SA‐β‐Gal staining revealed that 4,5‐diCQA significantly reduced cell senescence (Figure 7c). Thus, we selected 4,5‐diCQA for further study.

Molecular docking analysis indicated that 4,5‐diCQA can form hydrogen bonds with multiple amino acids of the human MYH9 protein (Figure 7d). Next, to verify whether 4,5‐diCQA can mitigate DPP4‐induced MYH9 upregulation, we added 4,5‐diCQA to both normal and DPP4‐overexpressing C28/I2 cells. The results showed that 4,5‐diCQA did not alter MYH9 expression in normal cells but downregulated MYH9 levels in DPP4‐overexpressing cells in a concentration‐dependent manner (Figure 7e,f). We hypothesized that 4,5‐diCQA might interfere with the interaction between DPP4 and MYH9. To test this hypothesis, we added 4,5‐diCQA to DPP4‐overexpressing C28/I2 cells. The results from IP and PLA assays showed that 4,5‐diCQA reduced the interaction between DPP4 and MYH9 (Figure 7g,h). Next, to determine whether 4,5‐diCQA promotes MYH9 ubiquitination, we added 4,5‐diCQA to both normal and DPP4‐overexpressing C28/I2 cells. The IP results showed that 4,5‐diCQA did not affect MYH9 ubiquitination in normal cells but significantly promoted MYH9 ubiquitination in DPP4‐overexpressing cells (Figure 7i.j).

To further determine whether 4,5‐diCQA reverses the harmful effects of DPP4 upregulation in chondrocytes, we treated C28/I2 cells under three conditions: transfected with a control plasmid (OENC), transfected with a DPP4OE plasmid, and transfected with a DPP4OE plasmid followed by treatment with 4,5‐diCQA. We then performed RNA‐seq analysis on these samples. Compared with chondrocytes transfected with the OENC plasmid, DPP4OE upregulated 2142 genes and downregulated 1099 genes. Additionally, in chondrocytes transfected with the DPP4OE plasmid, the addition of 4,5‐diCQA resulted in the downregulation of 210 genes and the upregulation of 259 genes (Figure 7k). We defined the genes that were upregulated by DPP4 and downregulated by 4,5‐diCQA as 4,5‐diCQA^Down^‐DEGs (99 genes). Conversely, we defined the genes that were downregulated by DPP4 and upregulated by 4,5‐diCQA as 4,5‐diCQA^Up^‐DEGs (41 genes) (Figure 7l). Pathway analysis revealed that the 4,5‐diCQA^Down^‐DEGs were primarily enriched in pathways related to inflammation and cell senescence. This indicates that 4,5‐diCQA can reverse the chondrocyte senescence and inflammation induced by DPP4 upregulation (Figure 7m). 4,5‐diCQA^Up^‐DEGs are enriched in metabolic pathways, including glutathione metabolism, which produces glutathione, an important antioxidant that protects cells from oxidative stress (Figure 7n). Arginine and proline metabolism are crucial for protein synthesis, and pyrimidine metabolism significantly impacts cell proliferation. This indicates that 4,5‐diCQA can counteract the adverse effects of DPP4 upregulation in chondrocytes by promoting cell proliferation and the biosynthesis of essential cellular components. In summary, these data demonstrate that 4,5‐diCQA can disrupt the interaction between DPP4 and MYH9, thereby promoting MYH9 ubiquitination and degradation. Additionally, 4,5‐diCQA can reverse the harmful effects caused by the upregulation of DPP4 in chondrocytes.

### 2.8 4,5‐diCQA Alleviates OA in Mice Induced by DMM and Aging

**Figure 8:**
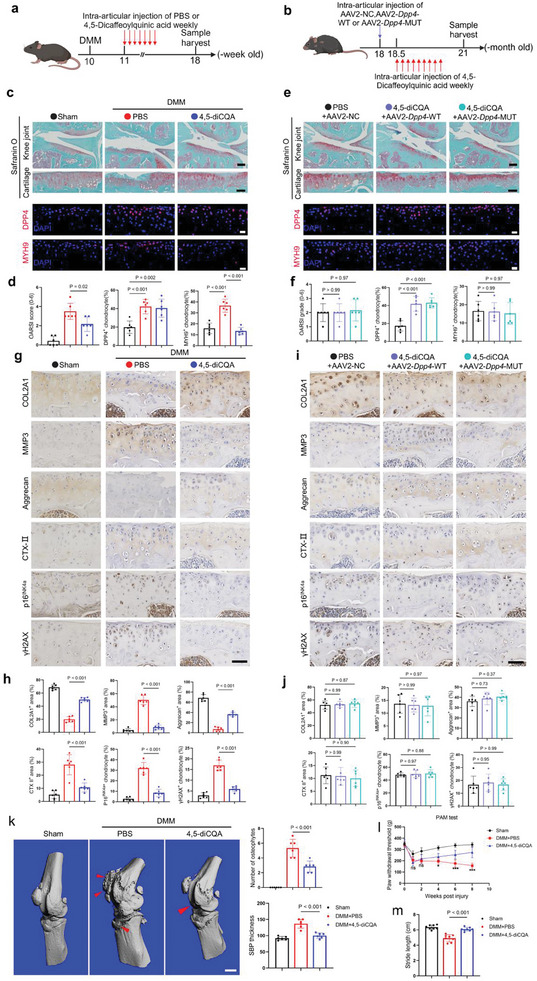
4,5‐diCQA alleviates DMM and aging‐induced OA progression in mice. a) Schematic diagram of the experimental design: 10‐week‐old mice underwent Sham or DMM surgery. One week after surgery, the DMM group received weekly intra‐articular injections of PBS or 4,5‐diCQA until 8 weeks after surgery, when knee joints were collected. b) Schematic diagram of the experimental design: 18‐month‐old mice received intra‐articular injections of AAV2‐NC, AAV2‐*Dpp4*‐WT, or AAV2‐*Dpp4*‐MUT. Two weeks later, AAV2‐NC mice received weekly PBS injections, while AAV2‐*Dpp4*‐WT and AAV2‐*Dpp4*‐MUT mice received weekly 4,5‐diCQA injections. Knee joints were collected 3 months later. c) Safranin‐O/fast green staining (scale bar = 250 µm) and IF (scale bar = 20 µm) analysis of knee joints from Sham, DMM+PBS, and DMM+4,5‐diCQA groups. d) Quantitative analysis of OARSI scores and the number of DPP4/MYH9‐positive cells from (c) (*n =* 6 mice per group). e) Safranin‐O/fast green staining (scale bar = 250 µm) and IF (scale bar = 20 µm) analysis of knee joints from PBS+AAV2‐NC, 4,5‐diCQA+AAV2‐*Dpp4*‐WT, and 4,5‐diCQA+AAV2‐*Dpp4*‐MUT groups. F) Quantitative analysis of OARSI scores and the number of DPP4/MYH9‐positive cells from (e) (*n =* 6 mice per group). g) IHC analysis of knee joints from Sham, DMM+PBS, and DMM+4,5‐diCQA groups (scale bar = 50 µm). h) Quantitative analysis of results from (g) (*n =* 6 mice per group). i) IHC analysis of knee joints from PBS+AAV2‐NC, 4,5‐diCQA+AAV2‐*Dpp4*‐WT, and 4,5‐diCQA+AAV2‐*Dpp4*‐MUT groups (scale bar = 50 µm). j) Quantitative analysis of results from (i) (*n =* 6 mice per group). k) Micro‐CT analysis of knee joints from Sham, DMM+PBS, and DMM+4,5‐diCQA groups, including quantitative analysis of osteophyte number and SBP thickness (scale bar = 1000 µm) (*n =* 6 mice per group). l) Knee joint pain assessment in Sham, DMM+PBS, and DMM+4,5‐diCQA groups (*n =* 8 mice per group). m) Stride length measurement in Sham, DMM+PBS, and DMM+4,5‐diCQA groups (*n =* 8 mice per group). One‐way analysis of variance (ANOVA) followed by Sidak correction for multiple comparisons is used for (d, f, h, j, k, and m). Two‐way analysis of variance (ANOVA) followed by Sidak correction for multiple comparisons is used for (l). Quantitative data are shown as mean ± s.d. Exact *p*‐values are shown in the figures. ***p < 0.001, *p < 0.05, NS, no significance.

To evaluate whether 4,5‐diCQA can alleviate OA in mice in vivo, we conducted weekly intra‐articular injections of PBS or 4,5‐diCQA. This was administered to 10‐week‐old mice one week after DMM surgery and to 18‐month‐old mice two weeks after injection with AAV2‐NC, AAV2‐*Dpp4*‐WT, or AAV2‐*Dpp4*‐MUT. For DMM‐induced OA, knee joints were collected 8 weeks post‐surgery. For aging‐induced OA, knee joints were collected 3 months after AAV2 injection (**Figure** **8**a,b). Safranin‐O/fast green staining analysis showed that mice treated with 4,5‐diCQA had lower OARSI scores compared to the PBS group. IF assay revealed that although 4,5‐diCQA could not alter the upregulation of DPP4 in chondrocytes post‐DMM surgery, it significantly reduced MYH9 expression compared to the PBS group (Figure 8c,d). For aging‐induced OA in mice, unlike previous results, AAV2‐*Dpp4*‐WT or AAV2‐*Dpp4*‐MUT did not increase the OARSI scores compared to the AAV2‐NC group (Figures 4e and 8e,f). IF analysis showed that AAV2‐*Dpp4*‐WT and AAV2‐*Dpp4*‐MUT still upregulated DPP4 expression in chondrocytes compared to AAV2‐NC. However, due to the presence of 4,5‐diCQA, MYH9 was not upregulated in chondrocytes in these two groups (Figure 8e,f). IHC results revealed that, compared to PBS, 4,5‐diCQA significantly upregulated the expression of COL2A1 and Aggrecan while downregulating the expression of MMP3, CTX‐II, p16^INK4a^, and γH2AX (Figure 8g,h). Similarly, for aging‐induced OA in mice, 4,5‐diCQA effectively prevented the DPP4‐induced downregulation of COL2A1 and Aggrecan, as well as the upregulation of MMP3, CTX‐II, p16^INK4a^, and γH2AX in both the AAV2‐*Dpp4*‐WT and AAV2‐*Dpp4*‐MUT groups (Figures 4i,j and 8i,j). Micro‐CT analysis showed that, compared to PBS, 4,5‐diCQA reduced osteophyte formation and alleviated subchondral bone sclerosis in the knee joints of mice following DMM surgery (Figure 8k). PAM tests indicated that 4,5‐diCQA increased the pain threshold in mice compared to PBS (Figure 8l). Gait analysis results demonstrated that 4,5‐diCQA effectively increased stride length and improved motor function in mice compared to PBS (Figure 8m; Figure S11, Supporting Information). In summary, these results supported that 4,5‐diCQA effectively alleviates the progression of DMM‐ and aging‐induced OA in mice.

## 3 Discussion

In this study, we investigated the pathological role of DPP4 in OA. Increased DPP4 expression was found in OA chondrocytes from both humans and mice. Overexpression of DPP4 promoted oxidative stress and cellular senescence in chondrocytes through excessive mitochondrial fission. At the molecular level, DPP4 competitively binds to MYH9, interfering with its E3 ubiquitin ligase CHIP, and resulting in upregulated MYH9, which promotes the recruitment of DRP1 to mitochondria via actin, causing excessive mitochondrial fission. Taken together, our results show that DPP4 exacerbates OA progression in an enzyme‐independent manner. Targeting this process through 4,5‐diCQA demonstrated alleviation in OA animal models.

The link between aging and OA is well established, but the causal mechanisms remain unclear. To adapt to ever‐changing environments, mitochondria finely regulate the balance between fusion and fission to repair mildly damaged parts and remove severely damaged ones.^[^ ^29^ ^]^ Previous studies have shown that excessive mitochondrial fragmentation can cause mitochondrial dysfunction by inducing mitochondrial DNA mutations, reducing ATP production, and generating excessive ROS, which further leads to cellular senescence.^[^ ^30^ ^]^ Similarly, in both human and mouse osteoarthritic cartilage, DRP1 expression is significantly elevated, leading to mitochondrial network disruption and chondrocyte apoptosis.^[^ ^25a^ ^]^ Alleviating excessive mitochondrial fission in vivo or in vitro in mice can mitigate chondrocyte apoptosis and degeneration.^[^ ^25^, ^31^ ^]^ Therefore, developing drugs that specifically target and mitigate excessive mitochondrial fission in senescent cells is crucial for the treatment of OA.

Through bioinformatics analysis and in vitro validation, we found that the DPP family exhibits conserved differential expression in osteoarthritic cartilage across human, mouse, and rat species. Recent reports showed that FAP, a DPP family member, is highly expressed in the articular cartilage, meniscus, and synovial tissue of OA patients, exacerbating OA progression by upregulating ECM degradation genes and directly degrading COL2A1.^[^ ^32^ ^]^ This is consistent with our findings in the present study. Additionally, previous studies have identified DPP4 as a key surface marker of senescent chondrocytes.^[^ ^14a^ ^]^ Intra‐articular injection of sitagliptin has been shown to ameliorate OA phenotype in rat DMM models.^[^ ^14b^ ^]^ However, these studies did not elucidate the mechanisms by which DPP4 induces chondrocyte senescence.

Recent studies have revealed that DPP4+ mesenchymal progenitor cell‐derived proteoglycan‐4 (Prg4^high^) fibroblasts are associated with the progression of OA.^[^ ^33^ ^]^ Our research showed that DPP4^high^ chondrocytes were more enriched in the cellular senescence pathway compared to DPP4^low^ chondrocytes. We hypothesize that DPP4+ chondrocytes may originate from DPP4+ mesenchymal progenitor cells. One possibility is that some DPP4+ mesenchymal progenitor cells differentiate into chondrocytes during development. Another possibility is that, during OA progression, some DPP4+ mesenchymal progenitor cells are activated and differentiate into DPP4+ chondrocytes.

In the current study, RNA‐seq, proteomics, and single‐cell sequencing analyses revealed that the upregulation of DPP4 in chondrocytes leads to the activation of oxidative stress and cellular senescence pathways. Additionally, we found that while DPP4 protein levels increase in osteoarthritic cartilage, its enzymatic activity does not change. This discrepancy may be explained by post‐translational modifications of DPP4 in osteoarthritic cartilage that prevent an increase in enzymatic activity. Previous studies have shown that glycosylation of DPP4 can downregulate its catalytic activity.^[^ ^34^ ^]^ Furthermore, we only assessed the enzymatic activity of the transmembrane DPP4 protein on the surface of chondrocytes, leaving the activity of soluble DPP4 protein unknown. Since DPP4 enzymatic activity did not change, our data suggest that the improvement in OA observed with sitagliptin may be independent of its inhibition of DPP4 enzymatic activity. For instance, sitagliptin can activate the p62–Keap1–Nrf2 signaling pathway to alleviate oxidative stress and excessive autophagy in acute lung injury, a mechanism that may also apply to OA improvement.^[^ ^35^ ^]^ Furthermore, through a series of gain‐of‐function experiments, we demonstrated that DPP4 induces excessive mitochondrial fission, leading to oxidative stress and cellular senescence in chondrocytes, independent of its enzymatic activity. Subsequently, through IP‐MS and proteomics analysis, we found that DPP4 binds to and upregulates MYH9 expression. MYH9 has been previously reported to promote mitochondrial fission via interactions with actin and DRP1.^[^ ^36^ ^]^ Additionally, we revealed that DPP4 binds to MYH9, interfering with CHIP‐mediated degradation and resulting in the accumulation of MYH9 in chondrocytes.

4,5‐diCQA is an isomer of diCQAs isolated from *Artemisia argyi*.^[^ ^37^ ^]^ It has strong antioxidant activity, α‐glucosidase, and α‐amylase inhibitory activity.^[^ ^38^ ^]^ Previous studies have shown it is effective in improving pancreatic and cognitive function in type 2 diabetes.^[^ ^37^, ^39^ ^]^ In this study, by virtual screening and in vitro validation, we demonstrated that 4,5‐diCQA can disrupt the interaction between DPP4 and MYH9, leading to a downregulation of MYH9 expression. RNA‐seq analysis showed that 4,5‐diCQA alleviates DPP4‐induced inflammatory responses and cellular senescence in chondrocytes while upregulating metabolic pathways. Importantly, 4,5‐diCQA exhibited therapeutic effects on both DMM‐ and aging‐induced OA in mice.

The current study elucidates the pathological mechanism by which DPP4 contributes to OA progression and identifies a natural compound, 4,5‐diCQA, that targets the interaction between DPP4 and MYH9, offering a new strategy for OA treatment. However, our study has a few limitations. Despite using AAV2 in animal experiments, which is safe and highly efficient for cartilage delivery, off‐target effects due to synovial cell infection are unavoidable. Additionally, the precise mechanisms by which the OA environment in vivo or IL‐1β in vitro induce DPP4 upregulation in chondrocytes remain unclear. Future studies are needed to further investigate why DPP4 enzymatic activity does not increase in OA chondrocytes despite the rise in DPP4 protein levels. What is more, whether DPP4 has other targets or other members of the DPP family play roles could provide a more profound understanding of OA pathogenesis.

## 4 Experimental Section

### Mice

C57BL/6 mice, aged 8 weeks and 18 months, were purchased from Dossy (Chengdu, China) and Qizhen (Hangzhou, China) laboratory animal companies, respectively. The mice were housed in a pathogen‐free environment with a 12 h light/dark cycle and had unrestricted access to food and water. All animal studies were approved by the Experimental Animal Ethics Committee of West China Hospital, Sichuan University (approval number: 20240527004). All experiments were conducted on male C57BL/6 mice due to previous studies indicating more severe OA in males following DMM surgery compared to females.^[^ ^40^ ^]^ Anesthesia was administered using isoflurane inhalation. For DMM‐induced OA, the medial meniscus ligament in the right knee of 10‐week‐old mice was cut to induce knee joint instability.^[^ ^41^ ^]^ In the sham‐operated group, only the joint capsule was incised without dissecting the medial meniscus ligament. Mice were euthanized 8 weeks after DMM or sham surgery, and the right knee joint was collected for further analysis. For age‐induced OA, 18‐month‐old mice were housed until they reached 21 months of age, at which point they were euthanized to obtain the right knee joint. The number of animals included in each experimental group was detailed in the figure legends.

### Intra‐Articular Administration

The pAAV2‐CMV‐MCS‐HA‐P2A‐eGFP (AAV2‐NC), pAAV2‐CMV‐*Dpp4*‐HA‐P2A‐eGFP (AAV2‐*Dpp4*‐WT), and pAAV2‐CMV‐*Dpp4*(S624A)‐HA‐P2A‐eGFP (AAV2‐*Dpp4*‐MUT) vectors were generated by WZ Biosciences Inc. (Jinan, China). 10 µL of AAV2 (1.0 × 10^12^ vg mL^−1^) was injected using a micro‐syringe (Hamilton, USA) into the right knee joints of mice either 2 weeks after DMM surgery or at the beginning of 18 months of age. Immunofluorescence experiments were performed to detect the expression of green fluorescent protein, confirming the successful infection of AAV2 (Figure S7a, Supporting Information). 4,5‐Dicaffeoylquinic acid (MCE, HY‐N0058) was intra‐articularly injected weekly at a dose of 0.5 µg/g body weight. The frequency and start time of the injections were indicated in the figure legends.

### Histological Analysis

Human cartilage tissue and mouse knee joints were fixed in 10% neutral formalin for 24 h, followed by decalcification in EDTA (pH 7.2) solution for 2 weeks. The decalcified tissues were embedded in paraffin, and the wax blocks were cut into 5 µm serial sections for Safranin‐O/fast green staining. Sections were collected when the meniscus of the medial compartment began to detach and stopped when the meniscus was no longer present in the tissue section, with ≈50 sections per sample. The degree of cartilage destruction was quantified by two observers under blinded conditions using the OARSI scoring system (grade 0–6) on sections stained with Safranin‐O/fast green stain.^[^ ^42^ ^]^ The Safranin‐O/fast green staining was performed using the Safranin‐O and Fast Green Stain Kit (Solarbio, G1371) following the manufacturer's protocol. The area used for calculating the OARSI score was the medial tibial plateau of the mouse knee.

### Human Cartilage Samples

Human articular cartilage was obtained from total knee replacement OA patients at the Department of Orthopaedics, West China Hospital, Sichuan University. The use of human specimens was approved by the Institutional Ethics Committee of West China Hospital, Sichuan University (No.201302007). The study included a total of 16 patients, with detailed information provided in Table S1, Supporting Information. The diagnosis of OA was based on X‐rays of the lower limbs. Cartilage from each patient was divided into intact (I) and damaged (D) sections according to the degree of damage.^[^ ^43^ ^]^ For protein and RNA extraction, cartilage samples were snap‐frozen in liquid nitrogen and stored at 80 °C. The remaining cartilage was fixed in a 10% neutral formalin solution for subsequent histological analysis.

### Behavioral Tests

OA‐associated pain was measured using the PAM device (Ugo Basile, 38500, Italy) at 1, 2, 4, 6, and 8 weeks after Sham/DMM surgery. The PAM device quantifies in real time the amount of force that causes pain in animals when pressing on a joint. Three days before the pain test, the mice were allowed to adapt to the environment and the sensation of grasping. The surveyor applied gradually increasing pressure to the right knee joint of the mice using a finger‐worn pressure transducer. The pressure was stopped when the mouse responded with a lower limb retraction, and the reading on the monitor was recorded as the lower limb contraction threshold.

Gait analysis was performed on mice 8 weeks after Sham/DMM surgery. The method involved allowing the mice to walk through a one‐way channel of appropriate width, with a camera positioned under the channel to record the footprints of the mice's front and back limbs. The footprints of different limbs were marked with different colors: dark blue for the left forelimb, red for the left hind limb, light blue for the right forelimb, and yellow for the right hind limb. Stride length was measured as the horizontal distance from the first time a particular foot touched the ground to the next time the same foot touched the ground.

### Cell and Cartilage Explant Culture

The C28/I2 human chondrocyte cell line was purchased from Sigma‐Aldrich (SCC043) and cultured in Dulbecco's modified Eagle's medium (DMEM)/F‐12 (Gibco) supplemented with 10% fetal bovine serum (FBS) and 1% penicillin/streptomycin. To upregulate the expression of *DPP4*, *MYH9*, *CHIP*, or ubiquitin in C28/I2 cells, plasmids were transfected using Lipofectamine 3000 (Invitrogen, L3000001) according to the manufacturer's instructions. Knockdown of *DPP4* and *CHIP* was achieved by transfecting shRNAs into C28/I2 cells using Lipofectamine 3000 following the manufacturer's protocol.

Primary mouse chondrocytes were isolated from the tibial plateau and femoral condyles of neonatal 3–5 day old mice as described previously.^[^ ^44^ ^]^ The translucent cartilage tissue at the knee joint was removed using forceps, cut into small pieces with a scalpel, and digested overnight in 0.5 mg mL^−1^ of collagenase D (Sigma‐Aldrich, 11088858001) at 37 °C. The cell digest was filtered using a 70 µm cell strainer, and chondrocytes were cultured in DMEM/F‐12 medium supplemented with 10% FBS and 1% penicillin‐streptomycin. Chondrocyte phenotyping was confirmed by observing the morphology under a light‐contrast microscope and examining the extracellular matrix using toluidine blue staining (Figure S1a, Supporting Information). The methods for upregulating *Dpp4* or *Myh9* and downregulating *Dpp4* in primary chondrocytes were similar to those used in C28/I2 cells. The sequences of shRNAs are listed in Table S2 (Supporting Information). The efficiency of transfection was assessed by Western blot analysis 48 h post‐transfection.

Mouse cartilage explants were isolated from the femoral heads of 8‐week‐old mice.^[^ ^45^ ^]^ Human cartilage explants were harvested using a 4‐mm biopsy punch from human cartilage samples.^[^ ^46^ ^]^ Both mouse and human cartilage explants were cultured in DMEM/F‐12 medium supplemented with 10% FBS, 1% penicillin‐streptomycin, and 1x insulin, transferrin, and sodium selenite (ITS, Gibco, 41400045). Lentiviruses, including pCDH‐CMV‐MCS‐EF1‐copGFP‐T2A‐Puro (OENC), pCDH‐CMV‐*DPP4*‐EF1‐copGFP‐T2A‐Puro (*DPP4*OE‐WT), pCDH‐CMV‐*DPP4*(S630A)‐EF1‐copGFP‐T2A‐Puro (*DPP4*OE‐MUT), pCDH‐CMV‐*Dpp4*‐EF1‐copGFP‐T2A‐Puro (*Dpp4*OE‐WT), and pCDH‐CMV‐*Dpp4*(S624A)‐EF1‐copGFP‐T2A‐Puro (*Dpp4*OE‐MUT), were generated by WZ Biosciences Inc (Jinan, China). To elevate the expression of DPP4, lentivirus, and polybrene (10 µg mL^−1^) (Solarbio, H8761) was added to the cartilage explant medium, with the medium changed every 2 days. The explants were collected on the tenth day after transfection and fixed in 10% neutral formalin. Cells and explants were cultured at 37 °C in an incubator with 5% CO2.

### Western Blot

Proteins were extracted from C28/I2 cells, primary chondrocytes, and human cartilage tissues using protein lysates (RIPA and protease inhibitor cocktail). Proteins were separated by sodium dodecyl sulfate‐polyacrylamide gel electrophoresis (SDS‐PAGE) and transferred to polyvinylidene fluoride (PVDF) membranes. The membranes were blocked with skim milk powder and then incubated with primary antibodies at 4 °C overnight. Details of the antibodies are listed in Table S3 (Supporting Information). The membranes were subsequently incubated with secondary antibodies, and signal detection was performed using an ECL substrate (Biosharp, BL520A).

### RT‐qPCR

TRIzol reagent (Invitrogen, 15596018CN) was used to extract RNA from cells and cartilage tissue. Reverse transcription reactions were performed using the Evo m‐MLV kit (Accurate Biology, AG11728) according to the manufacturer's instructions. The reactions were heated using a T100 Thermal Cycler (Bio‐Rad, 1861096). cDNA was amplified and detected using SYBR Green (Accurate Biology, AG11702) and QuantStudio3 (Applied Biosystems, A28567). Primers are listed in Table S4 (Supporting Information). Expression levels were normalized to GAPDH, and relative expression levels were calculated using the 2^−ΔΔCT^ method.^[^ ^47^ ^]^

### SA‐β‐Gal Staining

SA‐β‐Gal staining was performed using the Senescence β‐Galactosidase Staining Kit (Beyotime, C0602) according to the manufacturer's instructions. Cells were fixed with β‐galactosidase staining fixative for 15 min and then incubated with the staining solution overnight at 37 °C. Positively stained cells were counted in three different randomly selected fields within the same culture well.

### Immunofluorescence (IF) Staining

For cellular IF staining, cells were fixed with 4% paraformaldehyde and then permeabilized with 0.3% Triton X‐100 solution. Cells were blocked with normal goat serum and incubated overnight at 4 °C with primary antibodies. The next day, cells were incubated with secondary antibodies, including Goat Anti‐Rabbit IgG H&L (Alexa Fluor 488) (Abcam, ab150077) and Goat Anti‐Mouse IgG H&L (Alexa Fluor 594) (Abcam, ab150116), for 1 h, followed by incubation with DAPI to stain cell nuclei.

For tissue IF staining, the sections were baked in an oven at 60 °C for 2 h and dewaxed in xylene followed by a gradient series of alcohol solutions. The sections were then boiled in sodium citrate (pH = 6) solution for antigen retrieval, blocked with normal goat serum, and incubated with primary antibodies overnight at 4 °C. After incubation with secondary antibodies for 1 h, cell nuclei were stained with DAPI.

Images were observed and acquired using a confocal microscope (Zeiss, LSM710). Detailed information about the antibodies used is listed in Table S3 (Supporting Information). In each biological replicate, five images were selected, and three ROIs for statistical analysis.

### Immunohistochemistry (IHC)

The paraffin sections were baked at 60 °C for 2 h and then dewaxed with xylene and a series of gradient alcohol solutions. The sections were boiled in sodium citrate solution for antigen retrieval and incubated with hydrogen peroxide for 10 min to block endogenous peroxidase activity. After blocking, the sections were incubated with primary antibodies at 4 °C overnight. The next day, the sections were incubated with enzyme‐conjugated goat anti‐mouse/rabbit IgG polymerase at 37 °C for 20 min. Detection was performed using DAB (ZSGB‐BIO, ZLI‐9018), and the nuclei were counterstained with hematoxylin. The sections were then dehydrated with graded alcohol solutions and cleared in xylene. Images were captured using a slide scanner (Olympus VS200). Quantitative analysis of the proportion of stained positive cells and the area of stained positive regions in the images was performed using Image J software (version 1.53c, NIH). Details of the antibodies used are listed in Table S3 (Supporting Information). In each biological replicate, five images were selected, and three ROIs for statistical analysis.

### Mitochondrial Reactive Oxygen Species (ROS) Measurement

ROS were detected using the Reactive Oxygen Species Assay Kit (Beyotime, S0033S). Primary chondrocytes were transfected with plasmids, including pCDH‐CMV‐MCS‐EF1‐copGFP‐T2A‐Puro (OENC), pCDH‐CMV‐*Dpp4*‐EF1‐copGFP‐T2A‐Puro (*Dpp4OE*‐WT), and pCDH‐CMV‐*Dpp4*(S624A)‐EF1‐copGFP‐T2A‐Puro (*Dpp4*OE‐MUT). Then, the primary chondrocytes were incubated with 10 µmol L^−1^ DCFH‐DA at 37 °C for 20 min. The stained cells were then analyzed using a FACS Celesta flow cytometer (BD Biosciences) or observed under a confocal microscope (Zeiss, LSM710).

### JC1 and Mito‐Tracker Staining

Mitochondrial membrane potential was detected using the JC‐1 kit (Beyotime, C2006). Mitochondrial morphology analysis was conducted following the instructions of the Mito‐Tracker Red CMXRos kit (Beyotime, C1049B). Primary chondrocytes were transfected with OENC, *Dpp4*OE‐WT, and *Dpp4*OE‐MUT plasmids. Then, primary chondrocytes were incubated with the respective staining solutions for the JC‐1 and Mito‐Tracker assays. Images were then collected using a confocal microscope (Zeiss, LSM710).

### DPP4 Activity Assay

DPP4 enzyme activity in primary mouse chondrocytes and human cartilage tissue was measured using the DPP4 Activity Assay Kit (Abcam, ab204722). Samples were homogenized in DPP4 Assay Buffer and centrifuged at 13 000 g for 10 min to obtain the supernatant. A standard curve was generated by serially diluting 7‐Amino‐4‐Methyl Coumarin (AMC). The final volume of each sample in a 96‐well plate was adjusted to 50 µL with DPP4 Assay Buffer. The DPP4 Substrate was diluted 1:20 with DPP4 Assay Buffer to create the Reaction Mix. Then, 40 µL of the Reaction Mix was added to each well‐containing sample. The plate was incubated at 37 °C for 30 min. Fluorescence was measured using a microplate reader (TECAN, Spark) at excitation/emissio*n =* 360/460 nm at 10, 20, and 30 min. DPP4 activity was calculated using the formula provided in the kit's protocol.

### Immunoprecipitation (IP) and Mass Spectrometry (MS)

Cells were lysed on ice for 30 min using IP lysis buffer (Thermo, 87787), and the supernatant was collected after centrifugation. Anti‐HA beads (Thermo, 88836), anti‐FLAG beads (Thermo, A36797), or A/G agarose beads coated with the corresponding antibodies were added to the supernatant and incubated at 4 °C for 3 h. The beads were then washed three times with IP wash buffer (20 mм Tris‐HCl, pH 7.4; 274 mм NaCl; 0.5% NP‐40; 0.5 mм EDTA) and boiled with loading buffer for 10 min. The expression of the target protein was detected by western blot analysis.

To identify the target protein that binds to DPP4, C28/I2 cells were transfected with a control plasmid (OENC) or a DPP4‐HA plasmid. IP was then performed using HA beads, which were subsequently retained. MS experiments were conducted by PTO BIO (Hangzhou, China). Briefly, an appropriate amount of ammonium bicarbonate was added to the beads to adjust the pH to alkaline, followed by non‐contact ultrasonic lysis. Enzymatic digestion was performed using trypsin. The resulting peptides were dissolved in liquid chromatography mobile phase A and separated using the EASY‐nLC 1200 ultra‐high performance liquid chromatography system. Mobile phase A was an aqueous solution containing 0.1% formic acid and 2% acetonitrile; mobile phase B was an aqueous solution containing 0.1% formic acid and 90% acetonitrile. The flow rate was maintained at 500 nl/min. The ion source voltage was set to 2300 V, and the FAIMS compensation voltage (CV) was set to 70 V and 45 V. The peptide precursor ions and their secondary fragments were detected and analyzed using a high‐resolution Orbitrap mass spectrometer.

To identify the ubiquitination sites on MYH9, C28/I2 cells were co‐transfected with CHIP‐HIS, MYH9‐FLAG, and Ub‐MYC plasmids. After IP using FLAG beads, the magnetic beads were retained for further analysis. The secondary mass spectrometry data were then retrieved using Proteome Discoverer 2.4.

### Transmission Electron Microscopy

Cultured chondrocytes were centrifuged to form cell pellets, which were then fixed in electron microscopy fixative (Servicebio, G1102) for 2 h in the dark. Subsequently, the cell pellets were fixed in 1% osmic acid in 0.1 mol L^−1^ PBS for 2 h. After dehydration in graded ethanol, the cell pellets were embedded in epoxy resin. The sections were then prepared, and mitochondria were observed using transmission electron microscopy (Hitachi, HT7800).

### Micro‐CT

Before decalcification, the mouse knee joint was scanned using a SCANCO VivaCT80 cone‐beam scanner (10.5 µm resolution; 55 kVp source; 145 µA current). After image reconstruction, the number of osteophytes and the thickness of the subchondral bone of the medial tibial plateau were analyzed using 3D analysis in CT‐Analyser (Bruker).

### Proximity Ligation Assay (PLA)

Primary mouse chondrocytes were cultured in confocal glass dishes. The cells were fixed with 4% paraformaldehyde for 15 min and then washed three times with PBS. They were then permeabilized with 0.3% Triton‐X 100 solution for 3 min and washed three more times with PBS. Subsequent experimental steps were performed according to the manufacturer's instructions (Sigma, DUO92101).

### Molecular Docking and Virtual Screening

Molecular docking and virtual screening were performed by MedChemExpress. Due to the high homology between the drug‐binding regions of human and mouse MYH9, human MYH9 was chosen for docking with small molecule compounds. Given that the available three‐dimensional structure fragments of human MYH9 were too short, the ATP binding site (GLY174‐THR181) of the MYH9 structure predicted by AlphaFold (AlphaFold ID: AF‐P35579‐F1) was used for virtual screening. The structure of the human DPP4 protein was also predicted by AlphaFold (AF‐P27487‐F1). For the virtual screening, the following drug libraries were used: HY‐L001P MCE Bioactive Compound Library, PlusHY‐L901 50K Diversity Library, and HY‐L0055 V Discovery Diversity Set 50. Schrödinger Maestro 12.8 software was utilized for virtual screening, and PyMol was used for 3D visualization.

### RNA Sequencing (RNA‐seq) Analysis

RNA‐seq analysis was conducted by Personal Biotechnology (Shanghai, China) and LC‐Bio Technology (Hangzhou, China). Briefly, total RNA was extracted from C28/I2 cells transfected with OENC or DPP4‐OE plasmids using TRIzol reagent. The amount and purity of total RNA were assessed using NanoDrop ND‐1000 (NanoDrop, Wilmington, DE, USA). cDNA libraries were prepared using the NEB Next UltraTM RNA Library Prep Kit for Illumina (NEB, USA) and sequenced on the Illumina NovaSeq6000 platform. RNA‐seq was performed with the Illumina system, following protocols for 2 × 150 paired‐end sequencing.

Gene expression levels were quantified using featureCounts (v1.6.3), and differential expression analysis was performed with the DESeq2 R package (v1.20.0). P‐values were adjusted using the Benjamini–Hochberg method to control the false discovery rate (FDR). Gene expression differences were considered significant with | log_2_fold change | >0.5 and P‐value < 0.05. Gene Ontology (GO) and Kyoto Encyclopedia of Genes and Genomes (KEGG) pathway enrichment analyses were conducted using the clusterProfiler R package (v4.2.0).

Each KEGG pathway term and its associated genes were defined as gene sets. Gene Set Enrichment Analysis (GSEA) was performed on the Java GSEA platform (v3.0) using the “Signal2Noise” metric to generate a ranked list and the “gene set” permutation type. Gene sets with FDR values < 0.05 were considered statistically significant. The expression levels of leading genes in the enriched pathways identified by GSEA were visualized using heatmaps created with the ComplexHeatmap R package (v2.14.0).

### Single‐Cell Sequencing (scRNA‐seq) Data Processing

scRNA‐seq data were obtained from Gene Expression Omnibus (GEO) (GSE169454) and processed with the Seurat package in R software (v4.2.2). Chondrocytes from three human normal and four human OA cartilage samples were analyzed. Gene expression was normalized using sctransform. Principal component analysis (PCA) was performed on filtered variable genes with the RunPCA function in Seurat. The Harmony algorithm (v1.0) was employed to integrate single‐cell data from different platforms and batches, resulting in the integration of cells from ten patients using RunHarmony. The first 25 principal components were selected for t‐distributed stochastic neighbor embedding (t‐SNE), and Louvain clustering was conducted using FindClusters. Cell types were determined based on marker gene expression.

### 4D‐FastDIA Quantitative Proteomics

C28/I2 cells were transfected with either a control plasmid (OENC) or DPP4OE plasmid and then centrifuged to collect the cells. The samples were lysed by adding four volumes of lysis buffer and using ultrasound. The lysates were then centrifuged at 4 °C and 12 000 g for 10 min to remove cell debris. The supernatant was transferred to a new centrifuge tube for protein concentration determination using a BCA kit.

The samples were digested with trypsin, and the resulting peptides were dissolved in liquid chromatography mobile phase A. Peptide separation was performed using a NanoElute ultra‐high‐performance liquid chromatography system. The DIA data were processed using the DIA‐NN search engine (v.1.8). Tandem mass spectra were searched against the Homo_sapiens_9606_SP_20231220.fasta database (20429 entries) concatenated with a reverse decoy database. Trypsin/P was specified as the cleavage enzyme, allowing up to one missed cleavage. Fixed modifications included N‐terminal methionine excision and carbamidomethylation on cysteine. The FDR was set to <1%.

### Statistical Analysis

The sample size was established based on preliminary experiments and prior studies, taking into account the variability of the samples. Slide selection and quantification were performed in a randomized and blinded method. The statistical software used was GraphPad Prism (10.1.2). One‐way/Two‐way analysis of variance (ANOVA) followed by Sidak correction for multiple comparisons and Two‐tailed t‐tests were employed to determine statistical significance and P values. Data were presented as mean ± s.d.

### Ethics Approval

All animal studies were approved by the Experimental Animal Ethics Committee of West China Hospital, Sichuan University (approval number: 20240527004). The use of human specimens was approved by the Institutional Ethics Committee of West China Hospital, Sichuan University (No. 201302007).

## Conflict of Interest

The authors declare no conflict of interest.

## Author Contributions

X.L. and Z.Z. contributed equally to this work. X.L., Z.Z., and Z.H. conceptualized the study and revised the manuscript. X.L., Z.Z., Y.J., and W.J. performed the experiments and analyzed the data. X.L., Z.Z., W.G., and Z.H. interpreted the data. X.L. and Z.Z. prepared all the figures and tables. X.L., Z.Z., W.G.and Z.H. drafted the manuscript. W.G. and Z.H. performed critical revisions of the manuscript. All authors read and approved the final manuscript.

## Supporting information



Supporting Information

## Data Availability

The data that support the findings of this study are openly available in GEO at https://www.ncbi.nlm.nih.gov/geo/query/acc.cgi?acc=GSE273447 or https://www.ncbi.nlm.nih.gov/geo/query/acc.cgi?acc=GSE273261, reference number 273447 or 273261. The raw data for mass spectrometry and proteomics have been deposited in the ProteomeXchange consortium via iProX under accession code PXD054366.
