# Supplementary material for: Dipeptidyl Peptidase 4 (DPP4) Exacerbates Osteoarthritis Progression in an Enzyme‐Independent Manner
Source: Adv Sci (Weinh). 2024 Dec 16;12(6):2410525. doi: 10.1002/advs.202410525 (PMC11809337; doi:10.1002/advs.202410525)
Supplement: Supplementary file 1 — Supporting Information [file ADVS-12-2410525-s001.docx]

Supporting Information

**Dipeptidyl peptidase 4 (DPP4) exacerbates osteoarthritis progression in an enzyme-independent manner**

*Xinyu Li*, M.D.

*Zhao Zhang*, M.D.

*Wenyu Jiang*, M.D.

*Yucan Ju*, M.D.

*Weihua Guo*, PhD.^*^

*Zeyu Huang*, M.D., PhD.^*^

X. Li, Z. Zhang, W. Jiang, Y. Ju, Z, Huang

Department of Orthopaedic Surgery, Orthopaedic Research Institute, West China Hospital, West China Medical School

Sichuan University

Chengdu, 610041, China

Email: zey.huang@gmail.com

W. Guo

Department of Immuno-oncology

Beckman Research Institute at City of Hope

National Medical Center

Duarte, 91010, USA

Email: wguo@coh.org

**
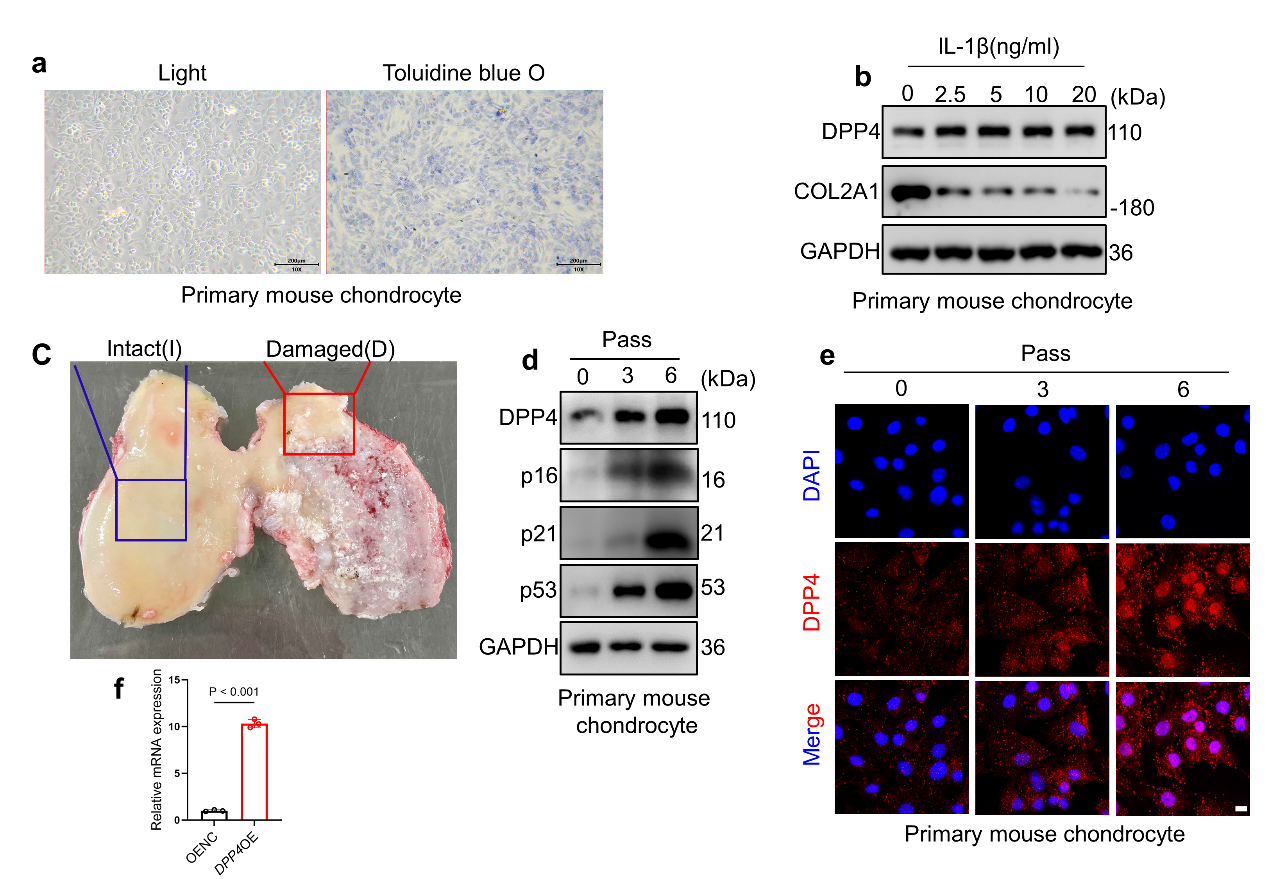
**

**Figure S1. Upregulation of DPP4 expression in senescent chondrocytes.** **a** Primary mouse chondrocytes incubated with Toluidine blue O dilutions (Scale bar=200μm). **b** Primary mouse chondrocytes were cultured for 24 h on dishes that were coated with 0, 2.5, 5, 10, 20 ng/ml IL-1β (n=3, biologically independent samples). **c** Intact (I) and damaged (D) articular cartilages from OA patients. **d, e** Western blot and IF (Scale bar=10μm) detection of DPP4 expression in passaged cells (n=3, biologically independent samples). **f** RT-qPCR analysis of DPP4 mRNA levels in C28/I2 cells transfected with *DPP4*OE or OENC plasmids (n=3, biologically independent samples). Two-tailed t-tests are used for (**f**) Quantitative data are shown as mean ± s.d. Exact p-values are shown in the figures.

**
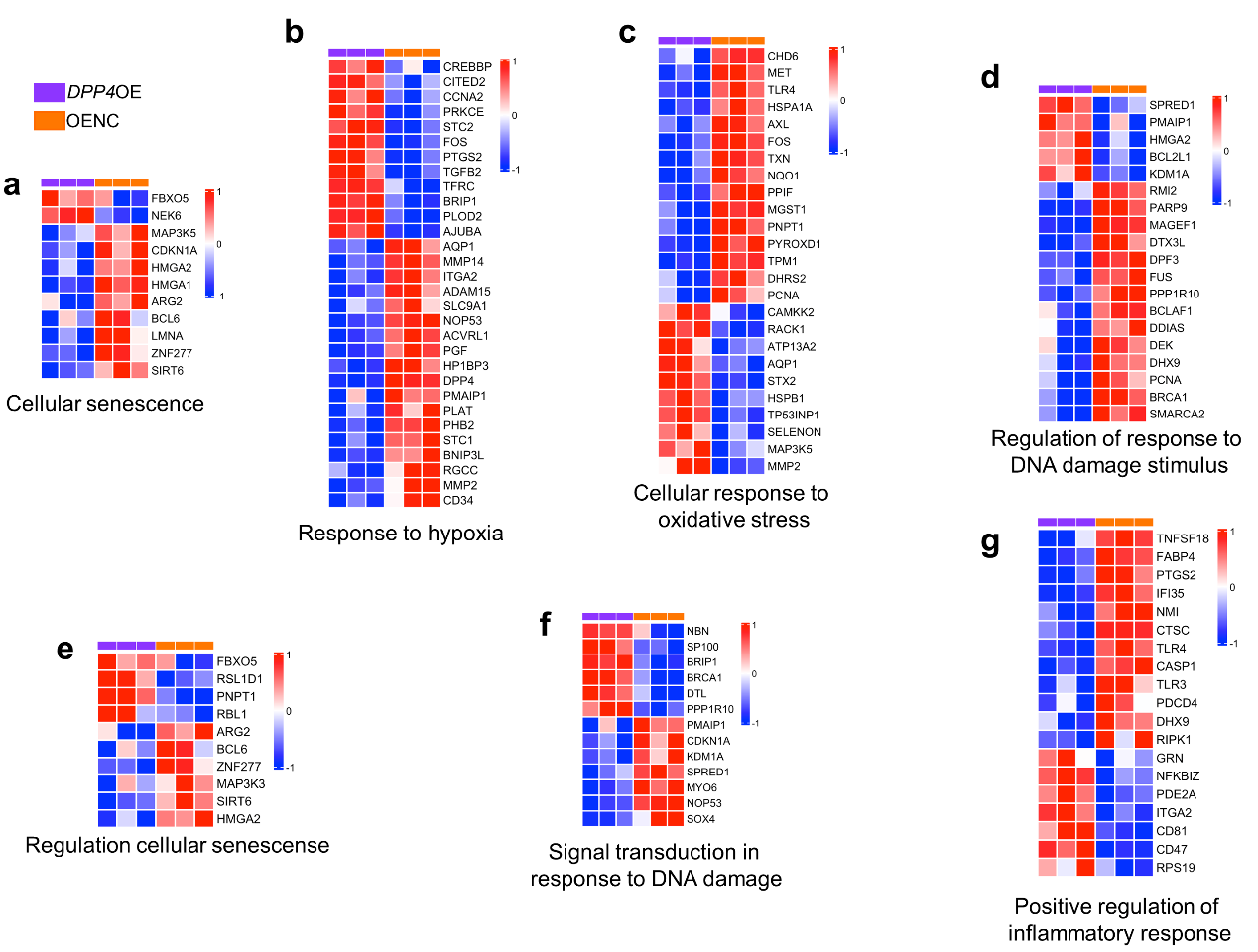
**

**Figure S2. The overexpression of DPP4 results in the upregulation of genes related to senescence and oxidative stress. Related to Figure 2**. **a-g** Heat map of gene expression (*DPP4*OE vs OENC) in the indicated pathway in C28/I2 cells.

**
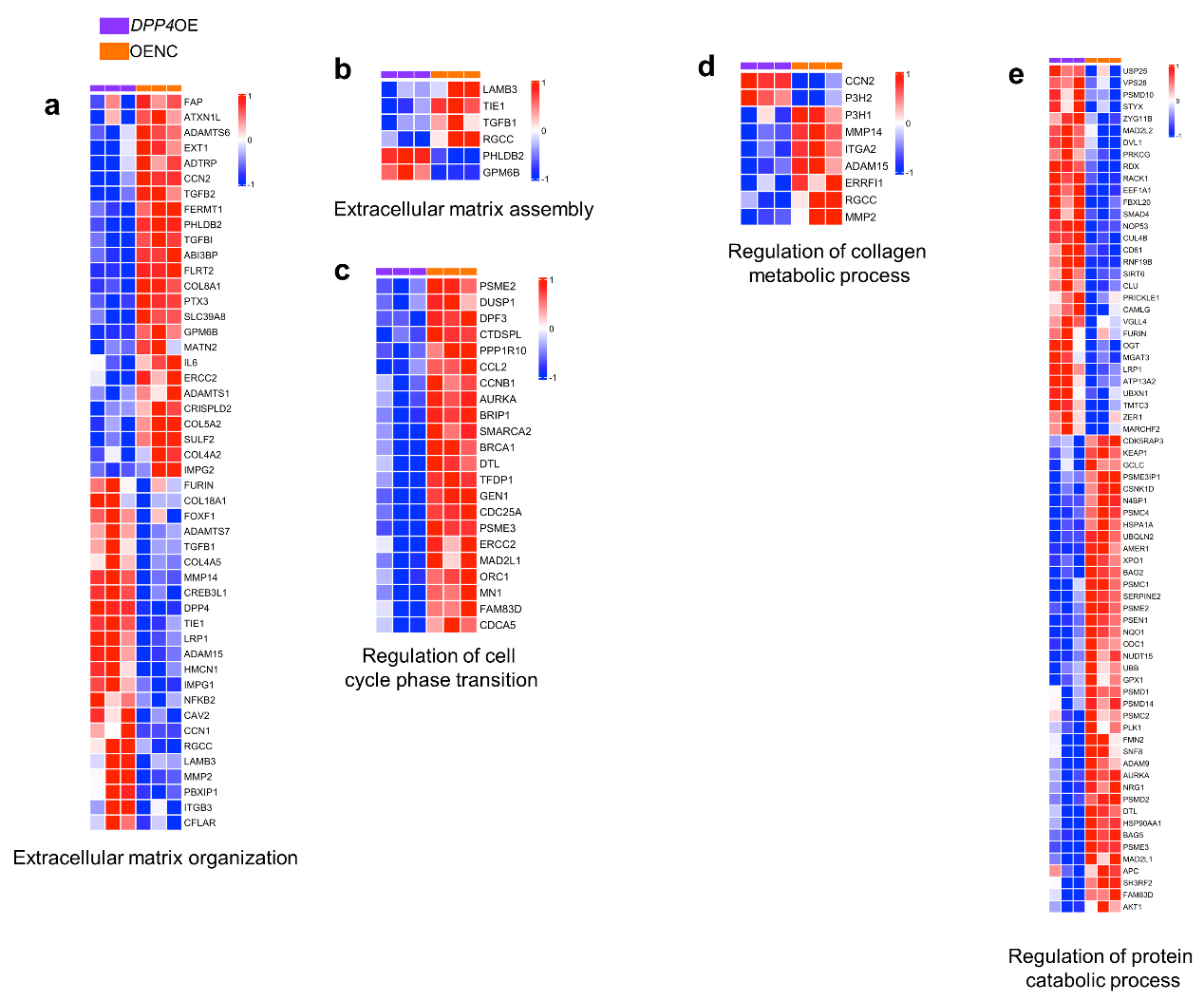
**

**Figure S3. The overexpression of DPP4 results in the downregulation of genes related to the collagen pathway and cell cycle. Related to Figure 2**. **a-e** Heat map of gene expression (*DPP4OE* vs OENC) in the indicated pathway in C28/I2 cells.

**
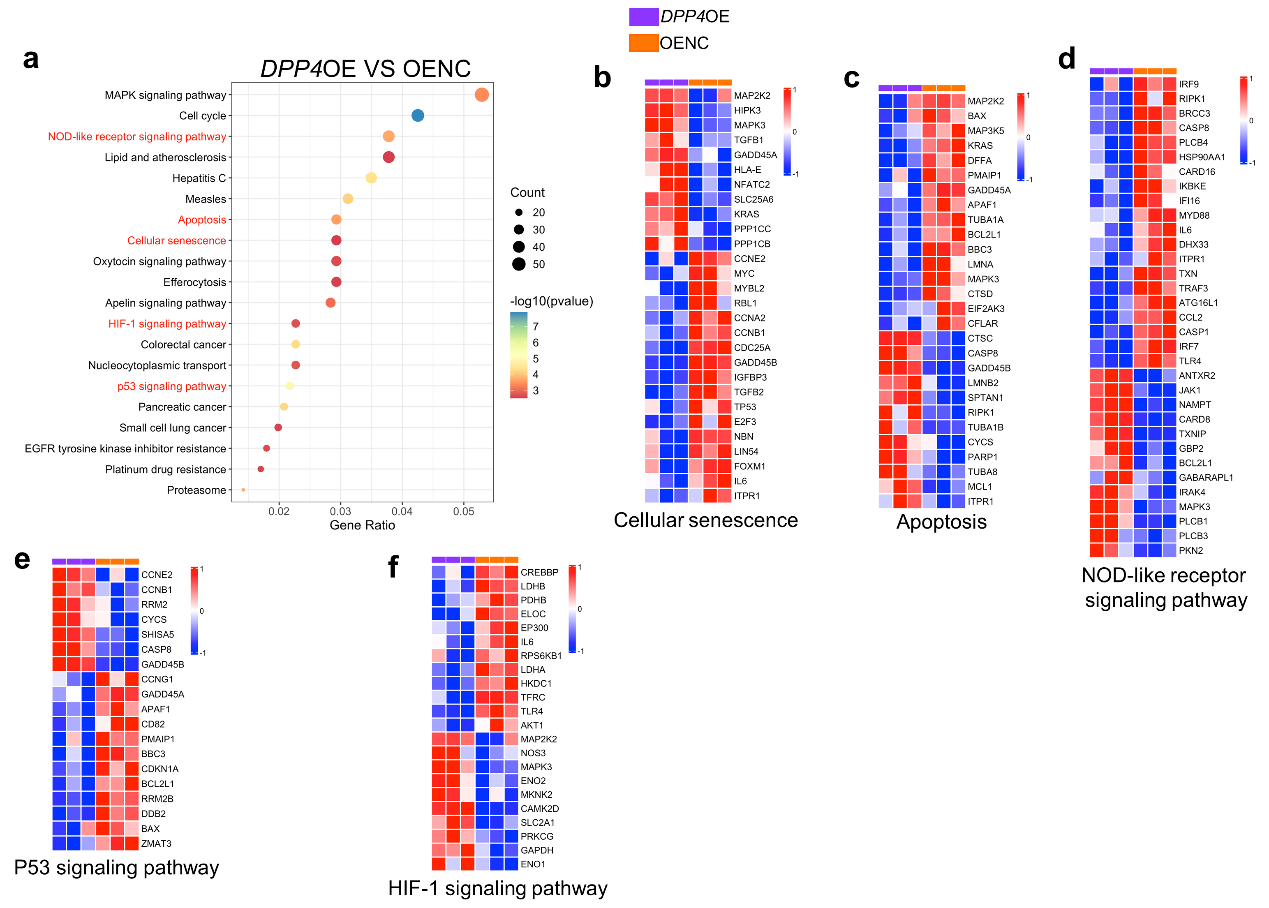
**

**Figure S4. The overexpression of DPP4 results in the differentially expressed genes. a** Enriched KEGG pathway analysis of differentially expressed genes (*DPP4*OE vs OENC) in C28/I2 cells. **b-f** Heatmap of gene expression (*DPP4*OE vs OENC) in the indicated pathway in C28/I2 cells.

**
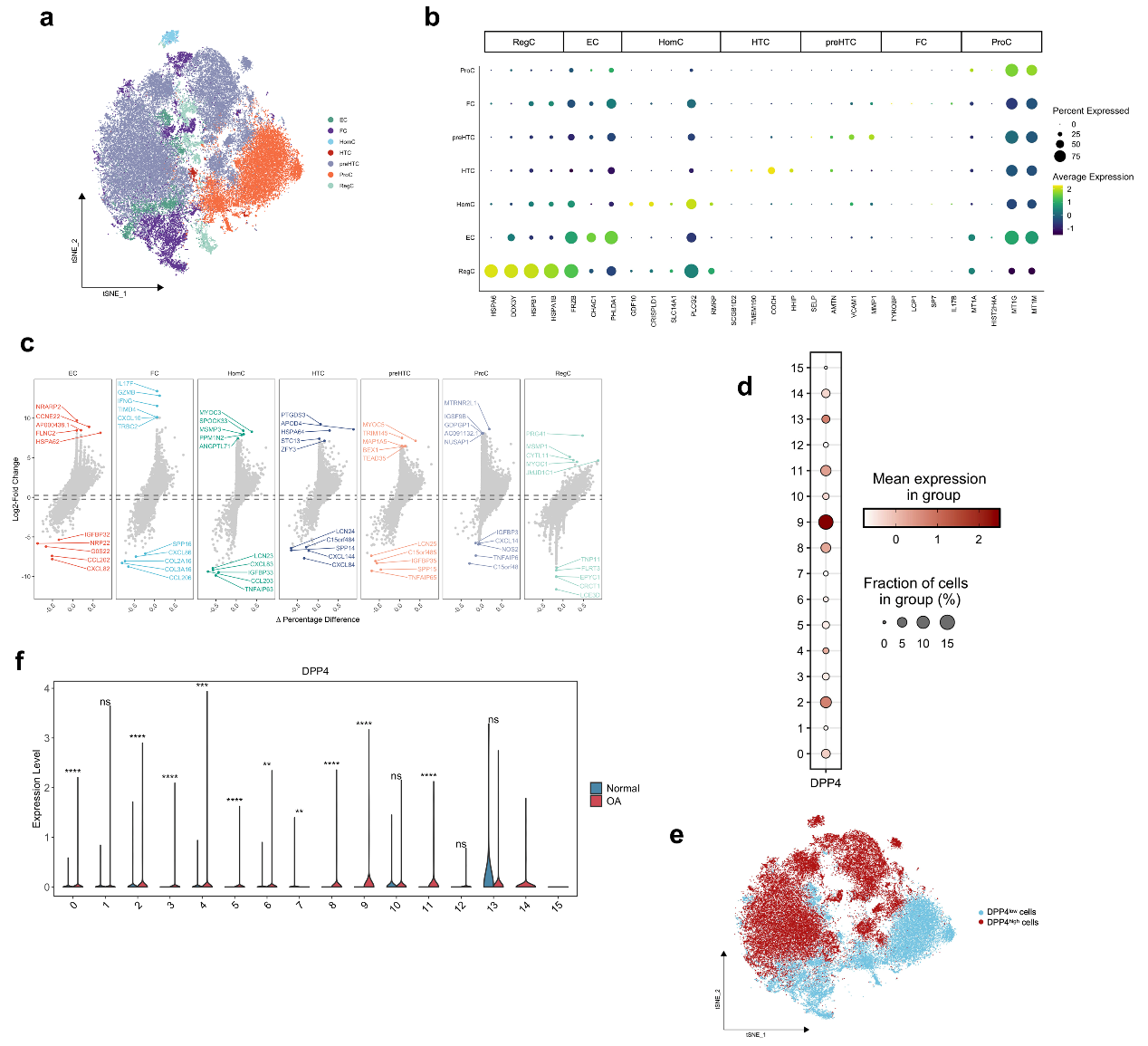
**

**Figure S5. Identification of chondrocyte populations and gene signatures in human OA and identification of subpopulations with high DPP4 expression.** **a** Single-cell RNA-seq was obtained from the GEO database (GSE169454). The UMAP plot of all cells was colored by their cell type identity. The classification of cell types is as follows: PreHTC (prehypertrophic chondrocyte), EC (effector chondrocyte), HTC (hypertrophic chondrocyte), RegC (regulatory chondrocyte), HomC (homeostatic chondrocyte), FC (fibrocartilage chondrocyte), ProC (proliferative chondrocyte). **b** The bubble chart shows some characteristic genes in different clusters. **c** Differential gene expression is represented by the dot plot. **d-f** Subgroups were clustered according to high or low DPP4 expression.

**
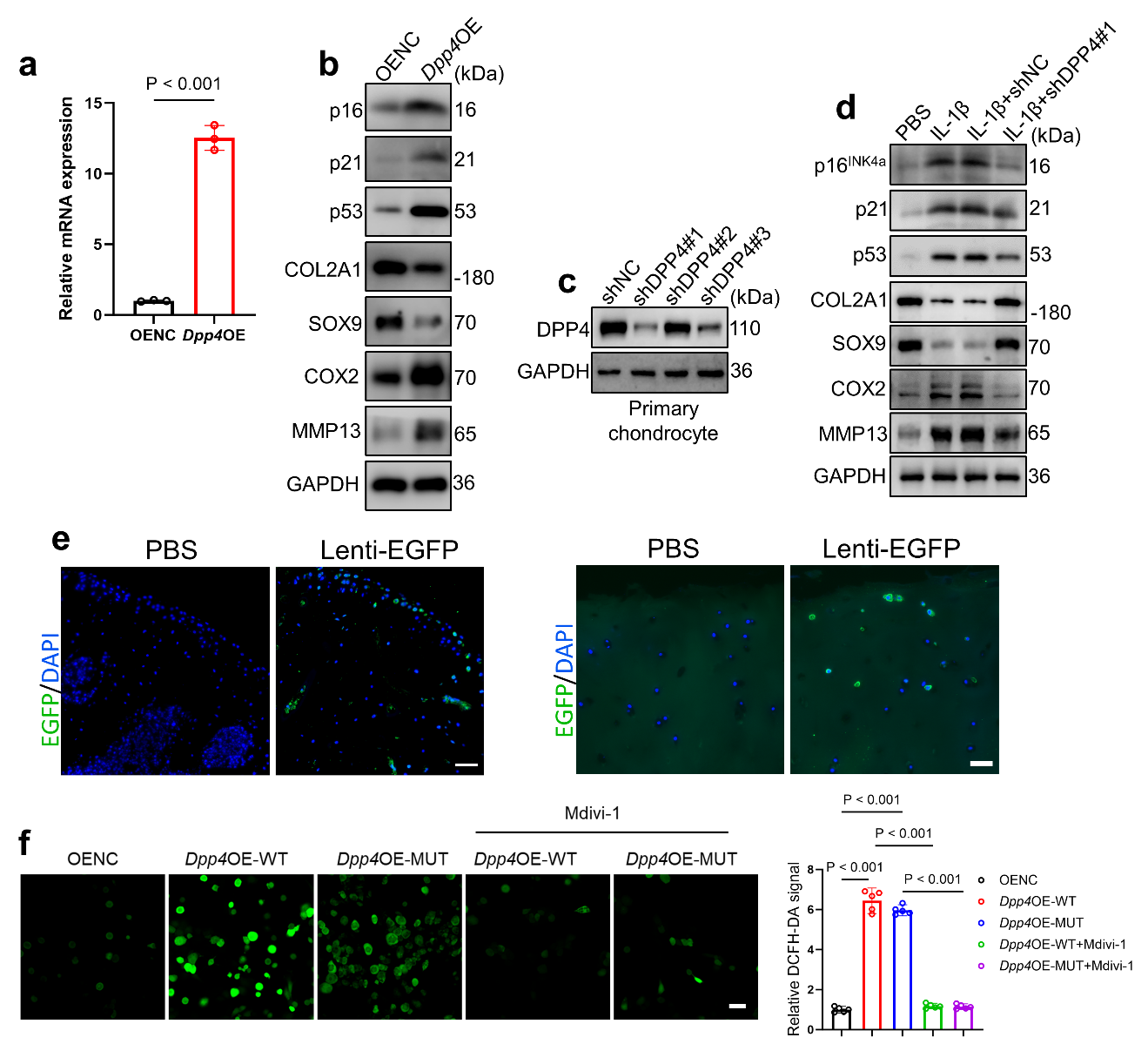
**

**Figure S6. DPP4 regulates aging and oxidative stress. a** Overexpressed DPP4 in primary mouse chondrocytes (n=3, biologically independent samples). **b** COL2A1, SOX9, COX2, MMP13, p53, p21, and p16^INK4a^ expression in primary mouse chondrocytes infected with OENC and *Dpp4*OE plasmids (n=3, biologically independent samples). **c** Western blot analysis confirmed the efficiency of DPP4 knockdown (n=3, biologically independent samples). **d** The expression of COL2A1, SOX9, COX2, MMP13, p53, p21, and p16^INK4a^ in primary chondrocytes transfected with either shNC or shDPP4 plasmids, with or without IL-1β stimulation (n=3, biologically independent samples). **e** After infecting the cartilage explants with Lenti-GFP, the GFP signal intensity in the explants was measured (Scale bar=50 μm). **f** DCFH-DA was used to detect ROS in primary chondrocytes transfected with *Dpp4*OE-WT or *Dpp4*OE-MUT plasmids, with or without the addition of Mdivi-1 (n=5, biologically independent samples). Two-tailed t-tests are used for (**a**) One-way analysis of variance (ANOVA) followed by Sidak correction for multiple comparisons is used for (**f**) Quantitative data are shown as mean ± s.d. Exact p-values are shown in the figures.

**
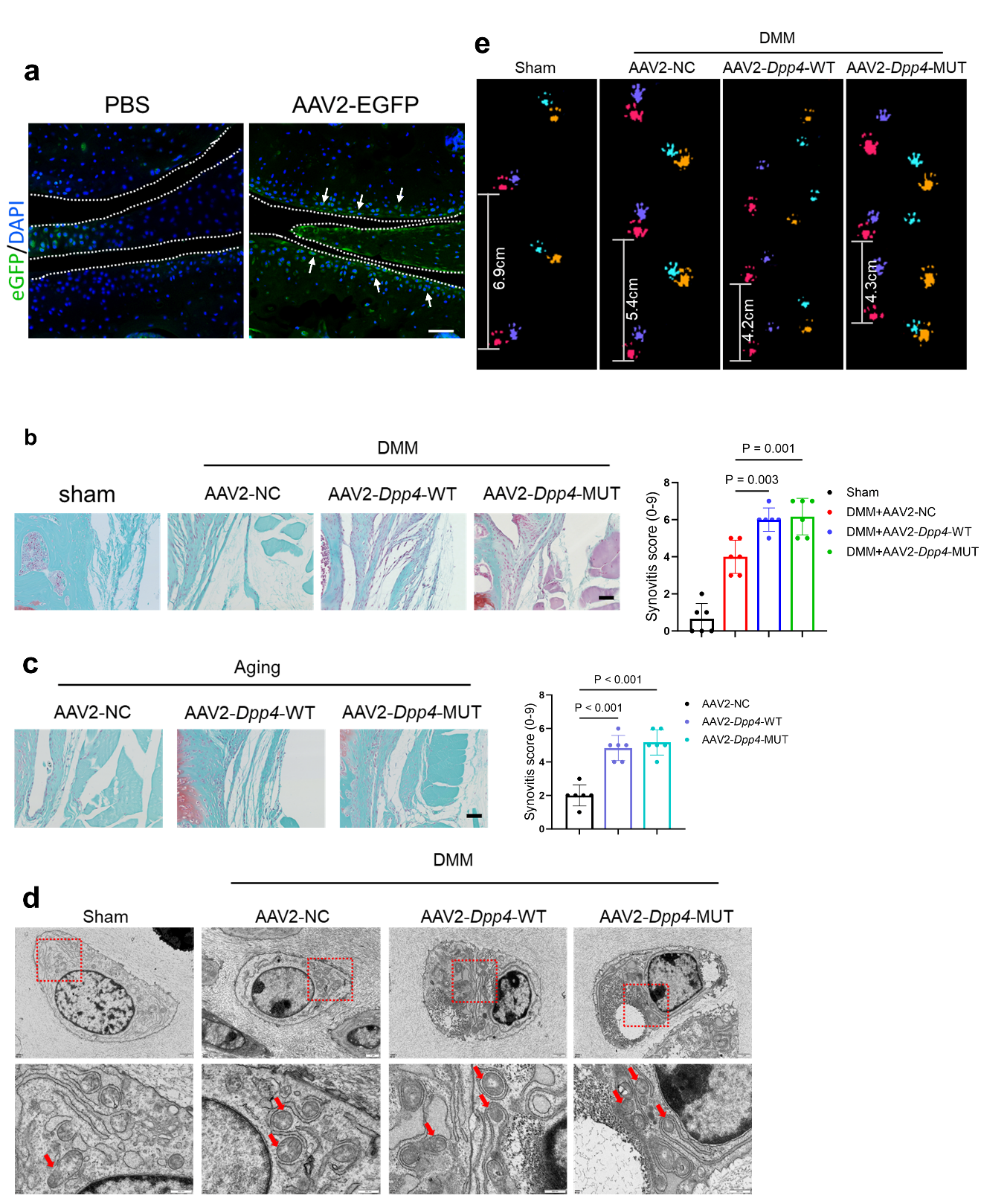
**

**Figure S7. Morphological and behavioral changes in mice after AAV2 injection a** Detecting the expression of GFP after injection of AAV2 (Scale bar = 50μm). **b, c** Synovial inflammation scores in DMM and aging-induced OA mice. **d** Transmission electron microscopy images of joint cartilage cells in DMM-induced OA mice (Scale bar = 1μm or 500 nm). **e** Gait analysis results in mice after DMM surgery and AAV2 injection. One-way analysis of variance (ANOVA) followed by Sidak correction for multiple comparisons is used for (**b, c**) Quantitative data are shown as mean ± s.d. Exact p-values are shown in the figures.

**
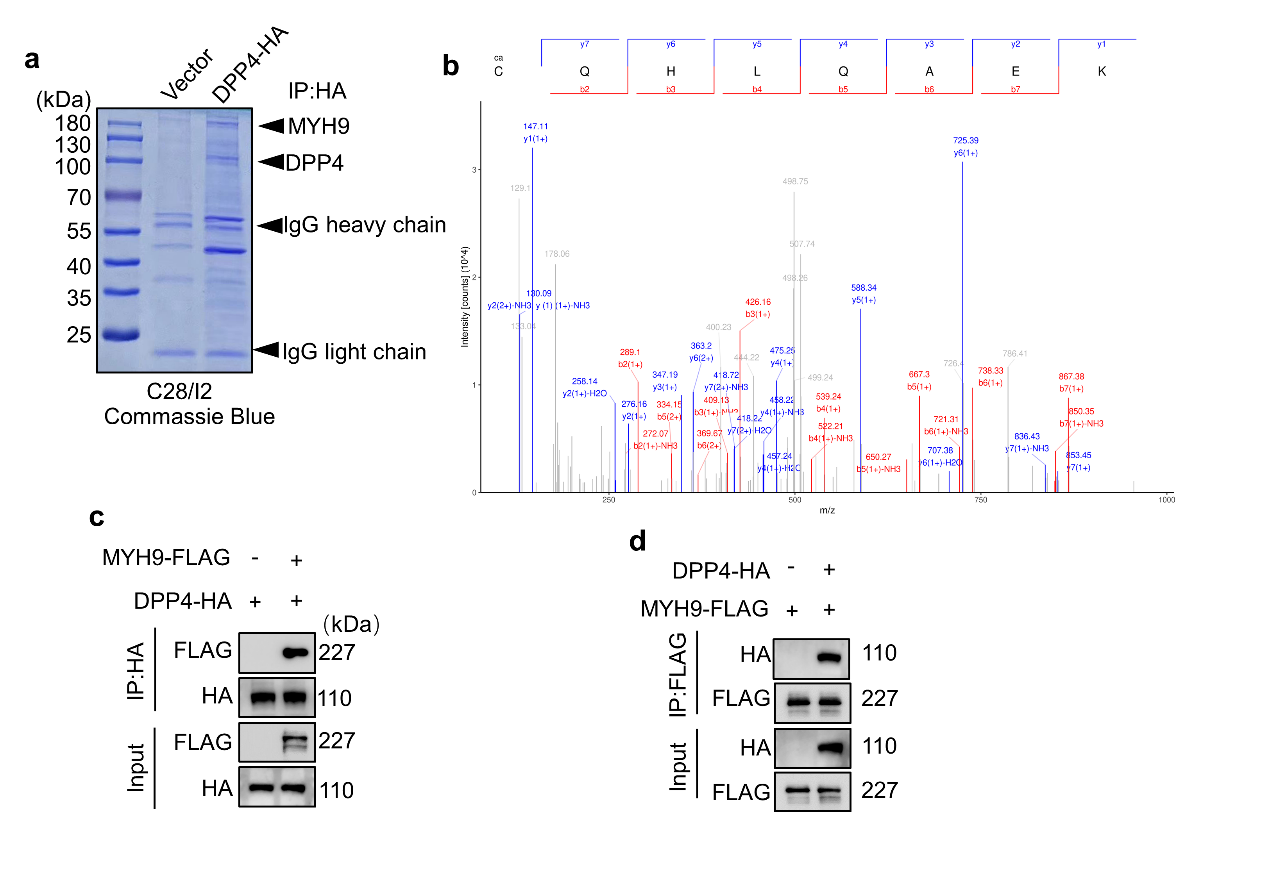
**

**Figure S8. DPP4 binds and upregulates MYH9 expression**. **a** Coomassie Blue stain of protein bound to DPP4. **b** Mass spectrometry identification of the binding protein MYH9 of HA-DPP4 immunopurified from HA magnetic beads. **c, d** DPP4 and MYH9 bind exogenously (n=3, biologically independent samples).

**
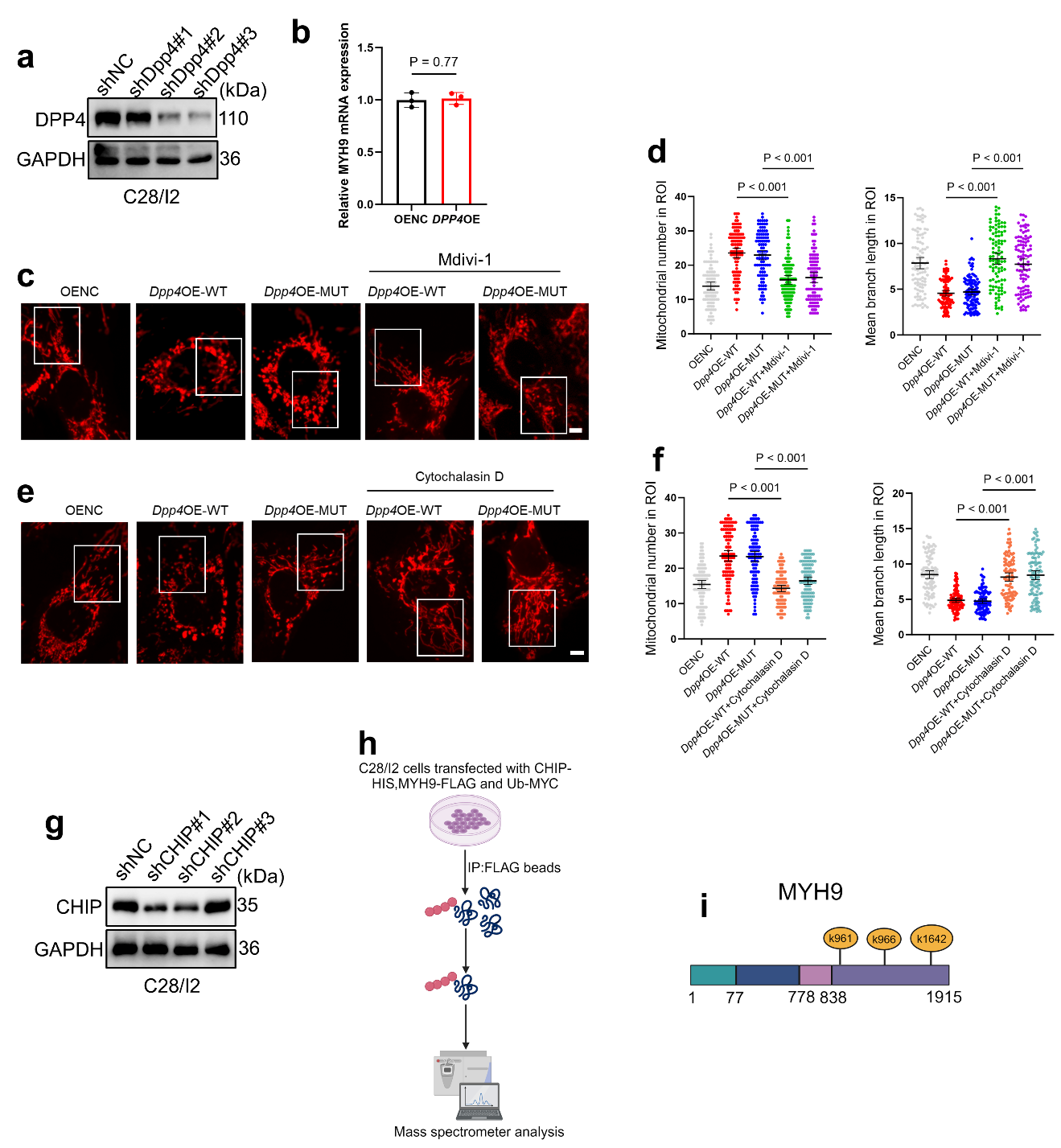
**

**Figure S9. DPP4 regulates mitochondrial fission through the DRP1/actin axis.** **a** Knockdown efficiency of DPP4 in C28/I2 cells transfected with three shDPP4 or control plasmids (n=3, biologically independent samples). **b** *MYH9* mRNA expression in C28/I2 treated with *DPP4*OE or control plasmids (n=3, biologically independent samples). **c, d, e, f** Primary chondrocytes were transfected with OENC, *DPP4*OE-WT, or *DPP4*OE-MUT plasmids and then treated with Mdivi-1 or Cytochalasin D, followed by MitoTracker staining and quantitative analysis (Scale bar=5μm) (n=5, biologically independent samples, 100 cells per group). For each sample, cells were randomly selected, and 15x20µm region was chosen as the ROI within each cell. **g** Knockdown efficiencies of CHIP in C28/I2 cells treated with three shCHIP or control plasmids (n=3, biologically independent samples). **h, i** Liquid chromatography-tandem mass spectrometry (LC-MS/MS) is performed to find out the ubiquitination site on MYH9. Two-tailed t-tests are used for (**b**) One-way analysis of variance (ANOVA) followed by Sidak correction for multiple comparisons is used for (**d, f**) Quantitative data are shown as mean ± s.d. Exact p-values are shown in the figures.

**
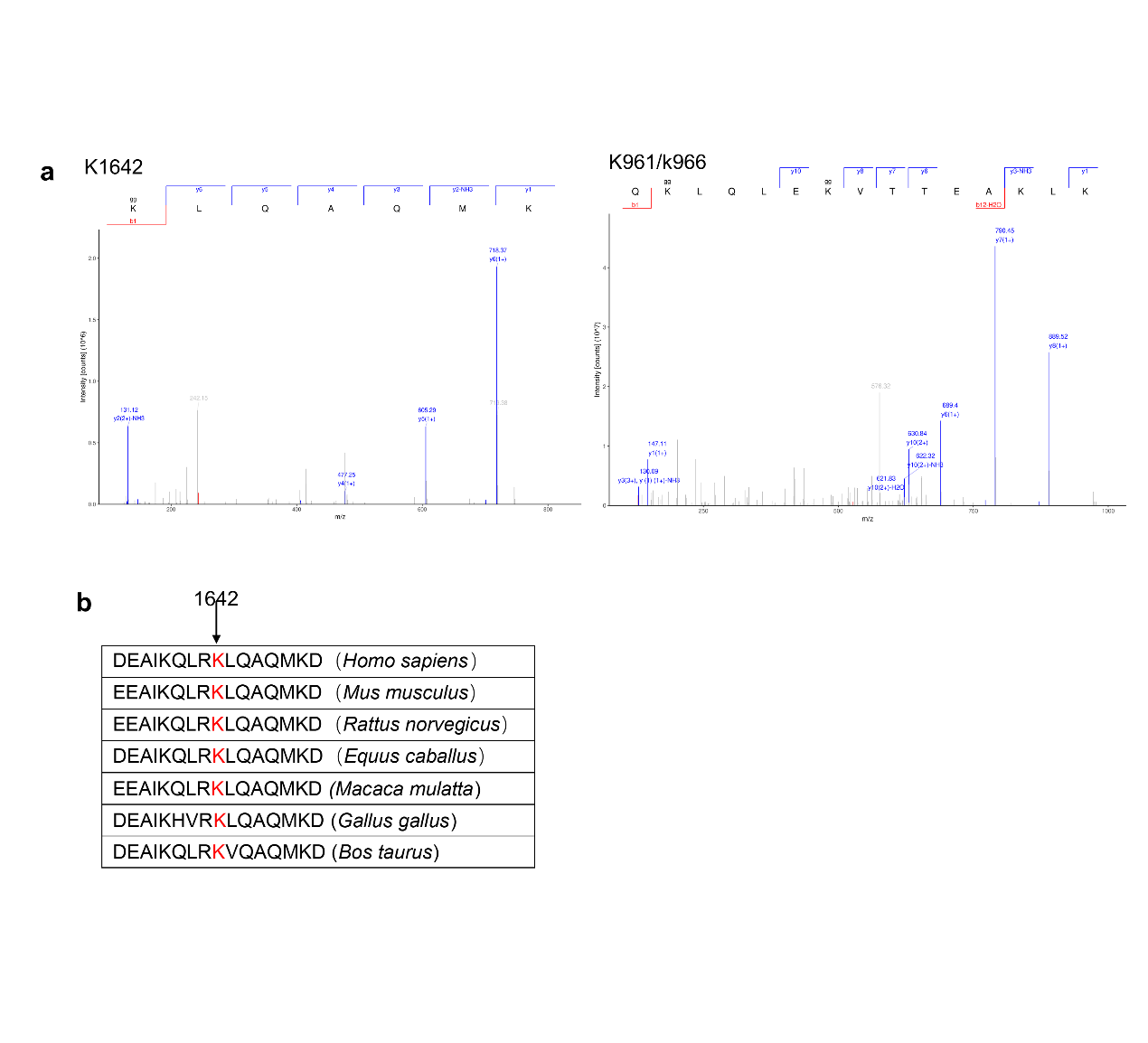
**

**Figure S10. K1642 as the major ubiquitination site of MYH9. a** The ubiquitination site on MYH9. **b** Conservation of the K1642 site on MYH9.

**
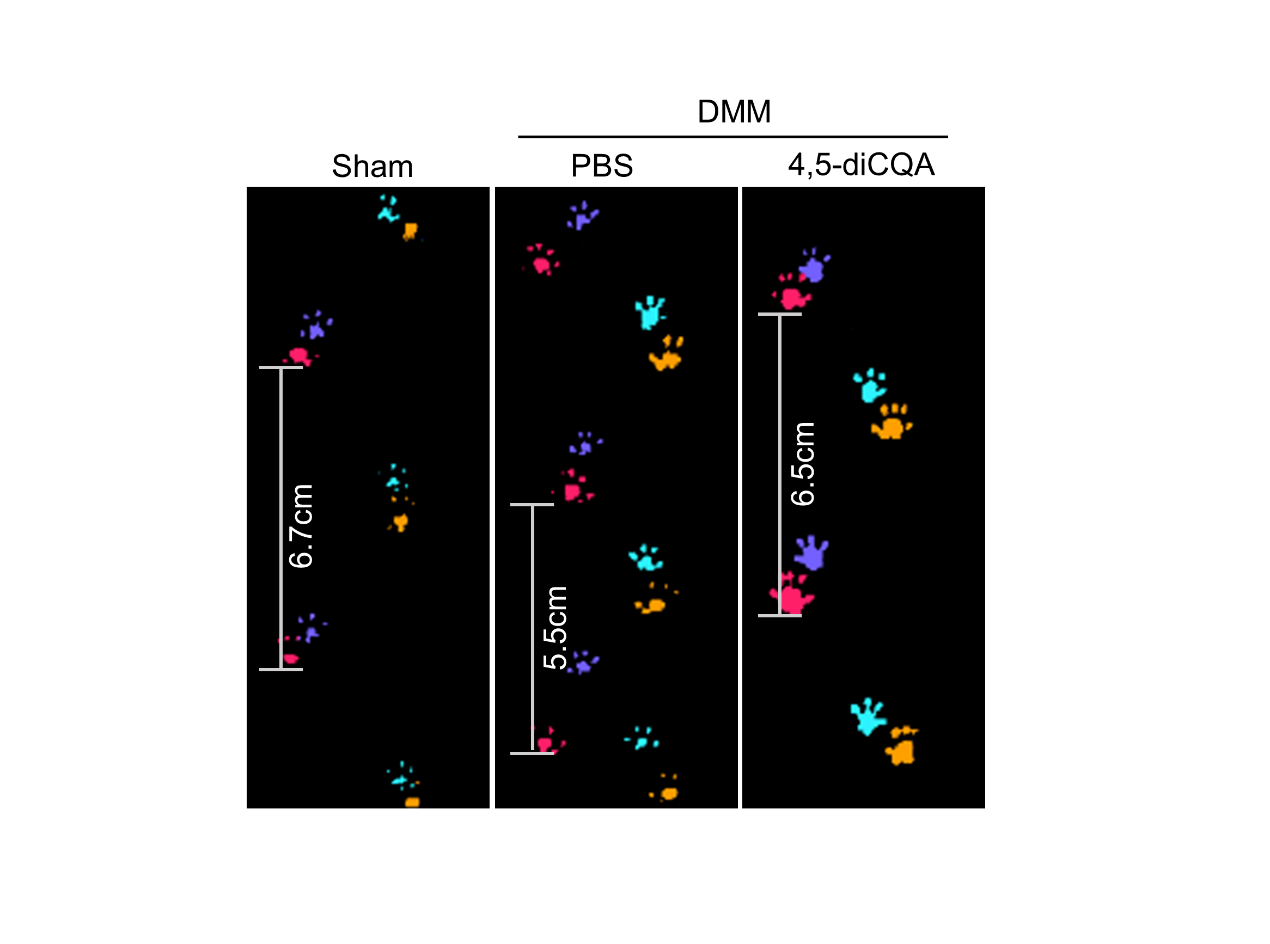
**

**Figure S11. Gait analysis demonstrates the effects of 4,5-diCQA in mice**. Stride length measurement is determined as the horizontal distance from the first ground contract of a foot to the next ground contact of the same foot.

**
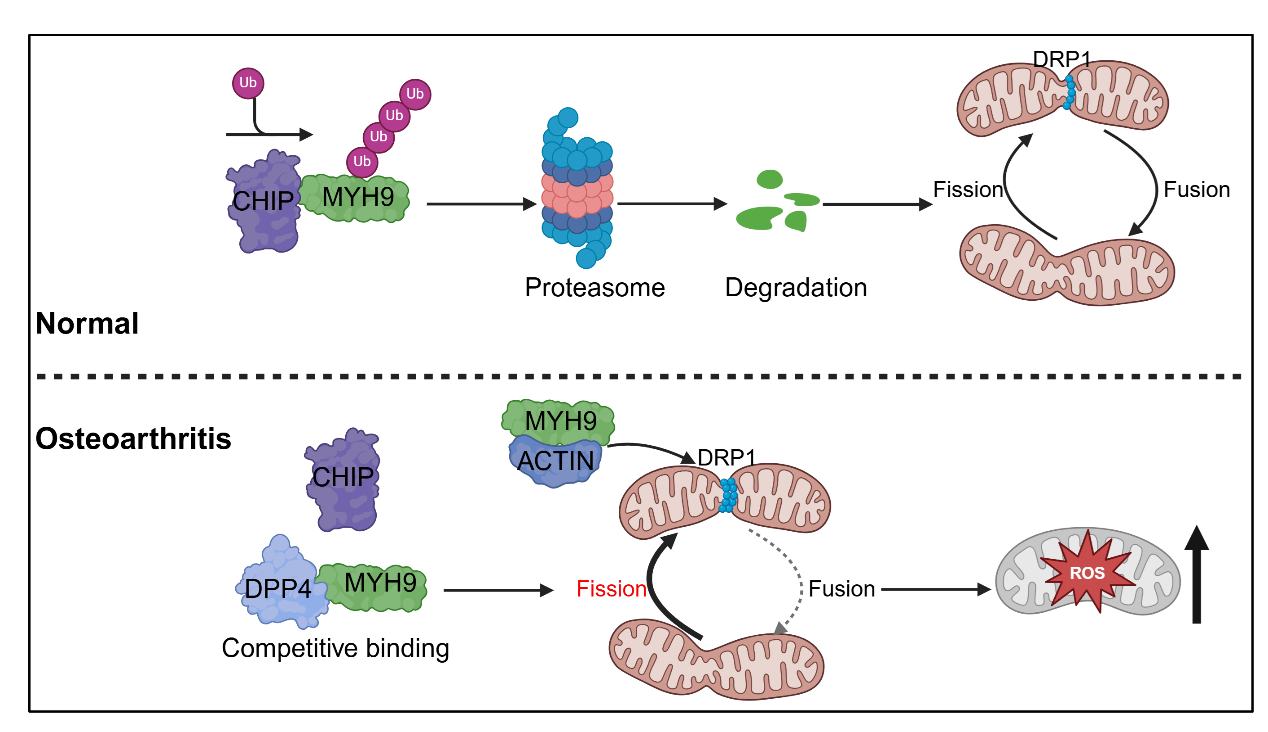
**

**Figure S12. Schematic diagram of DPP4-induced chondrocyte senescence and osteoarthritis progression.** Under normal conditions, CHIP mediates the degradation of MYH9 protein via the ubiquitin-proteasome pathway, maintaining MYH9 at normal levels and preserving the balance between mitochondrial fusion and fission. In OA, the expression of DPP4 is upregulated. DPP4 binds to MYH9 in an enzyme-independent manner, disrupting CHIP-mediated degradation of MYH9, leading to the accumulation of excess MYH9 in chondrocytes. MYH9 promotes the recruitment of DRP1 to mitochondria via actin, causing excessive mitochondrial fission. This results in mitochondrial dysfunction and the generation of large amounts of ROS. Elevated ROS levels induce oxidative stress, DNA damage, and cellular senescence in chondrocytes.

**Table S1.** **Detailed information of articular cartilage donor.**

| Sample | Age | Gender | KL | Extraction site |
| --- | --- | --- | --- | --- |
| 1 | 63 | M | 4 | Left femoral condyles |
| 2 | 59 | F | 3 | Left femoral condyles |
| 3 | 68 | F | 4 | Right femoral condyles |
| 4 | 63 | F | 3 | Left tibial plateau |
| 5 | 67 | M | 4 | Left tibial plateau |
| 6 | 69 | M | 4 | Right femoral condyles |
| 7 | 70 | M | 4 | Right tibial plateau |
| 8 | 60 | F | 3 | Right femoral condyles |
| 9 | 73 | M | 4 | Left femoral condyles |
| 10 | 79 | F | 4 | Left femoral condyles |
| 11 | 78 | F | 4 | Right femoral condyles |
| 12 | 73 | F | 3 | Right tibial plateau |
| 13 | 60 | F | 4 | Left femoral condyles |
| 14 | 64 | M | 4 | Right femoral condyles |
| 15 | 65 | F | 3 | Right tibial plateau |
| 16 | 58 | L | 4 | Left femoral condyles |

**Table S2.** **shRNA information**

| Gene | Sequence (5′→ 3′) |
| --- | --- |
| Mouse *Dpp4* | shRNA#1:GGAGAACAGTACCTTTGAAAGTTCAAGAGACTTTCAAAGGTACTGTTCTCCTTTTTT  shRNA#2:GCTGTGAATCCAACTGTAAAGTTCAAGAGACTTTACAGTTGGATTCACAGCTTTTTT  shRNA#3:GCTATCTGTGACTATGATAAGTTCAAGAGACTTATCATAGTCACAGATAGCTTTTTT |
| Human  *CHIP* | shRNA#1:CGCGAAGAAGAAGCGCTGGAATTCAAGAGATTCCAGCGCTTCTTCTTCGCGTTTTTT  shRNA#2:GCAGTCTGTGAAGGCGCACTTCTCGAGAAGTGCGCCTTCACAGACTGCTTTTTT  shRNA#3:GAAGAGGAAGAAGCGAGACATTTCAAGAGAATGTCTCGCTTCTTCCTCTTCTTTTTT |
| Human *DPP4* | shRNA#1:GGTCACCAGTGGGTCATAAATTTCAAGAGAATTTATGACCCACTGGTGACCTTTTTT  shRNA#2:GCACGGCAACACATTGAAATGTTCAAGAGACATTTCAATGTGTTGCCGTGCTTTTTT  shRNA#3:GCACAGCACACCAACATATATTTCAAGAGAATATATGTTGGTGTGCTGTGCTTTTTT |

**Table S3. Antibody information in this study.**

| Antibody | Brand | Application |
| --- | --- | --- |
| Rabbit anti-DPP4 | Abcam, ab215711 | WB (1:1000)  IF (1:200) |
| Rabbit anti-DPP4 | Abcam, ab187048 | WB (1:1000)  IF (1:200)  PLA(1:200) |
| Rabbit anti-MMP13 | Proteintech, 18165-1-AP | WB (1:1000)  IF (1:200) |
| Rabbit anti-COL2A1 | Proteintech, 28459-1-AP | WB (1:1000)  IF (1:200)  IHC (1:200) |
| Rabbit anti-p16INK4a | Abcam, ab211542 | WB (1:1000)  IF (1:200)  IHC (1:200) |
| Rabbit anti-p16INK4a | Proteintech, 10883-1-AP | IF (1:200) |
| Rabbit anti-Phospho-Histone H2A.X (Ser139) | CST,9718S | IF (1:200)  IHC (1:200) |
| Rabbit anti-p21 | Proteintech, 28248-1-AP | WB (1:1000) |
| Mouse anti-p53 | CST, 2524S | WB (1:1000) |
| Rabbit anti-Sox9 | CST, 82630S | WB (1:1000) |
| Rabbit anti-COX2 | Proteintech,12375-1-AP | WB (1:1000) |
| Mouse anti-GAPDH | Proteintech, 10494-1-AP | WB (1:5000) |
| Rabbit anti-OPA1 | Proteintech, 27733-1-AP | IF (1:200) |
| Rabbit anti-DRP1 | Proteintech, 12957-1-AP | IF (1:200) |
| Rabbit anti-MMP3 | Abclonal,A1202 | IHC (1:200) |
| Rabbit anti-Aggrecan | Proteintech, 13880-1-AP | IHC (1:200) |
| Rabbit anti-CTXⅡ | Cloud‐Clone Corp ,PAA686Mu01 | IHC (1:100) |
| Mouse anti-MYH9 | Proteintech, 60233-1-Ig | WB (1:2000)  IF (1:200)  PLA(1:200) |
| Rabbit anti-CHIP | Proteintech, 55430-1-AP | WB (1:1000)  IF (1:200)  PLA(1:200) |
| Rabbit anti-HA tag | Proteintech, 51064-2-AP | WB (1:5000) |
| Rabbit anti-DYKDDDDK tag | Proteintech, 20543-1-AP | WB (1:5000) |
| Mouse anti-6*His, His-Tag | Proteintech, 66005-1-Ig | WB (1:5000) |
| Rabbit anti-MYC tag | Proteintech, 16286-1-AP | WB (1:5000) |

**Table S4. Primer sequences used in qRT-PCR.**

| Gene | Primer Sequence (5′→ 3′) |
| --- | --- |
| Human *CTSC* | Forward primer: AGTATTTGCTGTTGGTTGTTTG  Reverse primer: TCCTTTCCCTATTGCTTCTTT |
| Human *DPP3* | Forward primer: CCTTCCATTTGTCAGCACTTT  Reverse primer: TGCCATTTCCTCATCTGGT |
| Human *DPP4* | Forward primer: GGGTCACATGGTCACCAGTG  Reverse primer: TCTGTGTCGTTAAATTGGGCATA |
| Human *DPP6* | Forward primer: GGCAAAATGAAGGAAAAGG  Reverse primer: CAAGGAGCAGATGACCAGA |
| Human *DPP7* | Forward primer: CGGTACTGCCTGGACACCT  Reverse primer: TCCCGTTGGAGAAGATGATG |
| Human *DPP8* | Forward primer: GACAGAGAGAAGAGGTGGTGA  Reverse primer: GGCAAGACAGTGATTTATTGATT |
| Human *DPP9* | Forward primer: CTACGACTTCCACAGCGAGA  Reverse primer: AGCACTGGGTCTTGATTTCC |
| Human *DPP10* | Forward primer: CTTAGACATTGGGCTTTACCA  Reverse primer: AGAGAACTTGAGGAGGACAC |
| Human *FAP* | Forward primer: CATCTGGAACTGGTCTTTTCA  Reverse primer: TTATCATCCTTTGTTGGGAGA |
| Human *PRCP* | Forward primer: GTAGAGTTCCTGGTTGCTTTTC  Reverse primer: TCTGCTTTTACTTTGTGTAGGGT |
| Human *PREP* | Forward primer: ACATCTACCAATCTCCACCAA  Reverse primer: CATCCATTTAGGTTCATCAGG |
| Human *GAPDH* | Forward primer:GGAGCGAGATCCCTCCAAAAT  Reverse primer: GGCTGTTGTCATACTTCTCATGG |
| Mouse *Tnf-α* | Forward primer: GACGTGGAACTGGCAGAAG  Reverse primer: CCACAAGCAGGAATGAGAAG |
| Mouse *Il-6* | Forward primer: GACTTCCATCCAGTTGCCT  Reverse primer: TAAGCCTCCGACTTGTFAA |
| Mouse *Mmp3* | Forward primer: GATGATGAACGATGGACAGA  Reverse primer: CCTTGGCTGAGTGGTAGAGT |
| Mouse *Mmp13* | Forward primer: TGTTTGCAGAGCACTACTTGAA  Reverse primer: CAGTCACCTCTAAGCCAAAGAAA |
| Mouse *Gapdh* | Forward primer: AGGTCGGTGTGAACGGATTTG  Reverse primer: GGGGTCGTTGATGGCAACA |
| Mouse *Dpp4* | Forward primer: CCGTGGAAGGTTCTTCTGGG  Reverse primer: GCTGCCGCTTCATCTTTGC |
